# The causal relevance of body mass index in different histological types of lung cancer: A Mendelian randomization study

**DOI:** 10.1038/srep31121

**Published:** 2016-08-04

**Authors:** Robert Carreras-Torres, Philip C. Haycock, Caroline L. Relton, Richard M. Martin, George Davey Smith, Peter Kraft, Chi Gao, Shelley Tworoger, Loïc Le Marchand, Lynne R. Wilkens, Sungshim L. Park, Christopher Haiman, John K. Field, Michael Davies, Michael Marcus, Geoffrey Liu, Neil E. Caporaso, David C. Christiani, Yongyue Wei, Chu Chen, Jennifer A. Doherty, Gianluca Severi, Gary E. Goodman, Rayjean J. Hung, Christopher I. Amos, James McKay, Mattias Johansson, Paul Brennan

**Affiliations:** 1 Section of Genetics, International Agency for Research on Cancer (IARC), Lyon, France; 2 MRC Integrative Epidemiology Unit, School of Social and Community Medicine, University of Bristol, Bristol, UK; 3 National Institute for Health Research Biomedical Research Unit in Nutrition, Diet and Lifestyle at University Hospitals Bristol NHS Foundation Trust and the University of Bristol, BS2 8AE, Bristol, UK; 4 Department of Epidemiology, Harvard T.H. Chan School of Public Health, Boston, USA; 5 Channing Division of Network Medicine, Brigham and Women’s Hospital and Harvard Medical School, Boston, USA; 6 Epidemiology Program, University of Hawaii Cancer Center, Honolulu, USA; 7 Norris Comprehensive Cancer Center, Keck School of Medicine, University of Southern California, Los Angeles, USA; 8 Roy Castle Lung Cancer Research Programme, The University of Liverpool Cancer Research Centre, Department of Molecular and Clinical Cancer Medicine, Institute of Translational Medicine, The University of Liverpool, Liverpool, UK; 9 Ontario Cancer Institute, Princess Margaret Cancer Center, Toronto, ON, Canada; 10 Genetic Epidemiology Branch, DCEG, National Cancer Institute, NIH, Rockville, USA; 11 Departments of Environmental Health and Epidemiology, Harvard T. H. Chan School of Public Health and Department of Medicine, Massachusetts General Hospital/Harvard Medical School, Boston, USA; 12 Program in Epidemiology, Division of Public Health Sciences, Fred Hutchinson Cancer Research Center, Seattle, USA; 13 Department of Epidemiology, Geisel School of Medicine, Dartmouth College, Lebanon, USA; 14 Human Genetics Foundation (HuGeF), Torino, Italy; 15 Fred Hutchinson Cancer Research Center, Seattle, USA; 16 Lunenfeld-Tanenbaum Research Institute of Mount Sinai Hospital, Toronto, Canada; 17 Department of Biomedical Data Science, Geisel School of medicine, Dartmouth College, Lebanon, USA

## Abstract

Body mass index (BMI) is inversely associated with lung cancer risk in observational studies, even though it increases the risk of several other cancers, which could indicate confounding by tobacco smoking or reverse causality. We used the two-sample Mendelian randomization (MR) approach to circumvent these limitations of observational epidemiology by constructing a genetic instrument for BMI, based on results from the GIANT consortium, which was evaluated in relation to lung cancer risk using GWAS results on 16,572 lung cancer cases and 21,480 controls. Results were stratified by histological subtype, smoking status and sex. An increase of one standard deviation (SD) in BMI (4.65 Kg/m^2^) raised the risk for lung cancer overall (OR = 1.13; P = 0.10). This was driven by associations with squamous cell (SQ) carcinoma (OR = 1.45; P = 1.2 × 10^−3^) and small cell (SC) carcinoma (OR = 1.81; P = 0.01). An inverse trend was seen for adenocarcinoma (AD) (OR = 0.82; P = 0.06). In stratified analyses, a 1 SD increase in BMI was inversely associated with overall lung cancer in never smokers (OR = 0.50; P = 0.02). These results indicate that higher BMI may increase the risk of certain types of lung cancer, in particular SQ and SC carcinoma.

Obesity increases the risk of many chronic diseases, including several cancers^1^, although observational studies have indicated an inverse relationship between body mass index (BMI), the most commonly used measure of obesity, and lung cancer risk, in particular among former and current smokers^2^,^3^,^4^,^5^,^6^,^7^,^8^. These associations may be due to a number of factors, in particular reverse causation (preclinical disease leading to weight loss). Smoking is also thought to reduce body weight, and residual confounding by tobacco smoking is another possible explanation for this inverse association. Given the strong increased risk of lung cancer caused by tobacco exposure and the well described association between tobacco consumption and reduced body weight^9^,^10^,^11^, traditional observational studies are unlikely to be able to fully account for the confounding effect of tobacco exposure when describing the relationship between BMI and lung cancer risk^9^,^10^,^11^.

Genetic epidemiology can circumvent some of the inherent limitations of observational epidemiology by modeling non-genetic risk factors using genetic instruments, and evaluating the association between the genetic instrument and disease risk rather than between the modifiable factor and disease risk. Germline gene variants are not affected by the presence of undiagnosed disease or associated with confounding exposures. Therefore, this technique, commonly referred to as Mendelian randomization (MR)^12^, is considered less sensitive to some of the biases that afflict traditional observational epidemiology^13^, in particular reverse causality and residual confounding. Our study utilized ‘two-sample Mendelian randomization’ to clarify the causal relationship between obesity and lung cancer risk, by constructing a genetic instrument for BMI in one study population, and subsequently evaluating the association of that genetic instrument with lung cancer risk in a large genome wide association study (GWAS)^13^.

## Materials and Methods

### Genetic instruments for BMI

Genetic instruments for BMI were identified using results from the Genetic Investigation of ANthropometric Traits (GIANT) consortium, a large collaborative GWAS on human body size and shape. Using GWA data on 339,224 individuals^14^, GIANT identified 97 single nucleotide polymorphisms (SNPs) independently (linkage disequilibrium R^2^ < 0.1) associated with BMI at a genome-wide significance level. In the GIANT study population, these 97 SNPs explained 2.7% of in-study variance in BMI. For each of the 97 SNPs, we retrieved the effect estimate that was expressed in standard deviations (SD) of BMI (SD change in BMI per-allele, β_GP_), along with the relevant standard error from the consortium website, and coded each SNP so that the reference allele was associated with an increase in BMI^15^. In the GIANT study population, 1 SD change in BMI equaled 4.65 kg/m^2^.

### Lung cancer, phenotypic BMI and tobacco related data

Summary statistics on lung cancer risk, including OR estimates and standard errors for instrumental SNPs, were available from the Transdisciplinary Research In Cancer of the Lung (TRICL) and International Lung Cancer Consortium (ILCCO)^16^ based on 4 lung cancer GWAS with a total of 11,348 lung cancer cases and 15,861 controls. Individual level data were available from three sources, *i)* a subset of the TRICL GWAS including 2,554 lung cancer cases and 3,825 controls from the International Agency for Research on Cancer-ILCCO sample (IARC-ILCCO)^16^, *ii)* 1,437 lung cancer cases and 1,453 controls from the European Prospective Investigation into Cancer and Nutrition (EPIC) study^17^ who had been genotyped using the GAME-ON OncoArray^18^, and *iii)* 3,456 lung cancer cases and 3,850 controls based on 7 studies genotyped using a custom Affymetrix Axiom Array (Affymetrix, Santa Clara, CA, USA)^19^. Only European descent subjects were included in the study. Each study has been specifically approved by the Ethical Committees of the original studies and all the participants provided a written informed consent. Covariates available from the IARC-ILCCO, EPIC, and Axiom data (namely measured BMI, smoking status, cigarettes per day, and additional measures of historical smoking exposure such as pack-years) were used to validate the genetic instrument based on the GIANT study, as well as to evaluate if the BMI genetic instrument was associated with measures of tobacco exposure. All genome-wide studies were imputed using the 1000 Genomes Project ALL panel (Phase I integrated Release 3) in the original projects. Imputation quality parameters were checked in each lung cancer sample for the 97 BMI-instrumental SNPs, and only SNPs with imputation quality higher than 0.6 were selected for the Mendelian randomization analyses.

### Statistical methods

To evaluate the validity of the genetic instruments, we initially constructed a genetic score for BMI with individual allele dosages weighted by the estimated per-allele effect on BMI (β_GP_) as provided by the GIANT consortium^14^. The association between the BMI genetic score and measured BMI was subsequently evaluated in each of the studies where individual level data were available. We similarly evaluated the association between the BMI instrument and measures of tobacco exposure (cigarettes per day (CPD), cotinine levels, and pack-years (PY)). These relationships were modeled using linear regression, controlling for age, sex and principal components to account for population stratification. Pooled estimates of the association between BMI genetic score and the tested BMI and smoking phenotypes were obtained by fixed-effects meta-analysis using the *metagen* R package, and I^2^ statistic to quantify the proportion of the total variation due to heterogeneity were calculated. Additionally, power calculations for the MR analysis were performed acording to Burgess^20^, assuming a nominal statistical significance of alpha 0.05 and a genetic instrument explaining 2.7% of phenotype variance^14^.

To evaluate the association between the BMI instrument and lung cancer risk, SNP to disease effects (β_GD_) were obtained by meta-analyses of the different study effect estimates using the METAL software^21^. The overall causal effect of BMI on lung cancer risk was subsequently estimated using a likelihood-based approach^22^. Since the BMI instrument was calibrated in units of SD of BMI (4.65 kg/m^2^ in the GIANT consortium), the resulting OR and 95% confidence interval provide an estimate of relative risk of lung cancer caused by a one SD increase in BMI.

To evaluate the extent to which the risk estimates may be driven by pleiotropy, we re-evaluated the association between the BMI instrument after excluding the rs11030104 SNP which was reported to be associated with smoking initiation by the GIANT consortium^14^. In addition, we used two complementary approaches: sensitivity analyses for the likelihood approach to possible departures of the main assumption of an absence of pleiotropy, namely the weighted median estimator^23^ and the MR-Egger approach^24^. The weighted median estimator is the median of a distribution in which Wald ratio estimates (β_GD_/β_GP_) have been ordered and represent percentiles of this distribution. The percentile which each ratio estimate represents is given by a weighting formula as a function of the standardized inversed variance of the ratio estimates^23^. The MR-Egger approach performs a weighted linear regression of the SNP to disease effects (β_GD_) on the SNP to phenotype effects (β_GP_), giving evidence for potential overall directional pleiotropy^24^.

## Results

### Analyzed samples

The total sample, with available GWAS data or summary risk association estimates, comprised 21,480 controls and 16,572 lung cancer cases overall. From those cases with histology information available, there were 5,282 adenocarcinoma (AD), 4,224 squamous cell (SQ) and 904 small cell (SC) cases. For samples with individual level data, the proportion of ever smokers among control groups ranged from 65 to 68%, while among the cases the rates were higher, between 89 and 94%. Within histology groups, AD cases presented lower proportions of ever smokers (82–86%) compared with SQ and SC cases (96–98%). Likewise, the distribution of sex was not uniform among histological groups. The proportion of males in AD cases ranged from 43 to 64%, while in SQ and SC cases the range was between 52 and 87% (Supplementary Table S1). After excluding SNPs with low imputation quality, the number of contributing SNPs was 92 for TRICL and IARC-ILCCO, 96 for EPIC and 65 for Axiom data sets. Further information on each of the 97 SNPs is presented in Supplementary Tables S2–S5.

### Validation of BMI instrument

**Figure 1:**
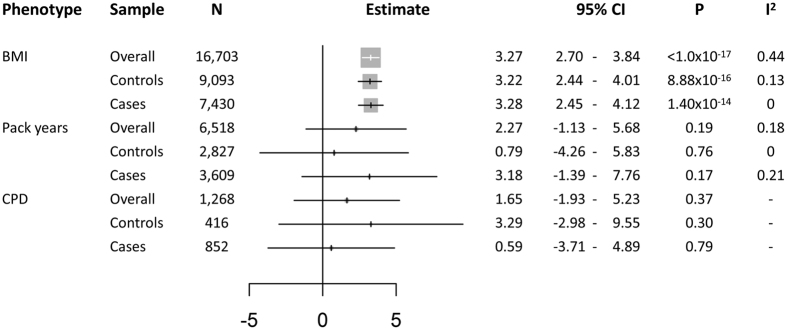
Forest plot of association analyses between genetic BMI score and measured BMI, pack years, and cigarettes per day (CPD) in the whole sample and within cases and controls. Cotinine levels are shown in Supplementary Table S6. 95%CI: 95% Confidence Interval; P: P value. I^2^: Heterogeneity coefficient.

The BMI instrument was associated with measured BMI in our study sample (Change in BMI kg/m^2^ per unit of the genetic score [95%CI] = 3.27 [2.70–3.84]; P < 1.0 × 10^−17^), and was not associated with available measures of tobacco exposure (P > 0.19), including pack years, cigarettes per day (CPD) or cotinine levels (Fig. 1). Association results within each study for the BMI score instrumental validation are shown in Supplementary Table S6. Power calculations indicated that our sample provided sufficient statistical power (80%) to detect an OR of 1.21 for overall lung cancer, 1.32 for adenocarcinoma, 1.36 for squamous cell carcinoma, 1.85 for small cell carcinoma, 1.36 for lung cancer overall ever smokers, and 2.14 for lung cancer overall never smokers (Supplementary Fig. S1).

### BMI causal effect estimation using a likelihood-based MR approach

**Figure 2:**
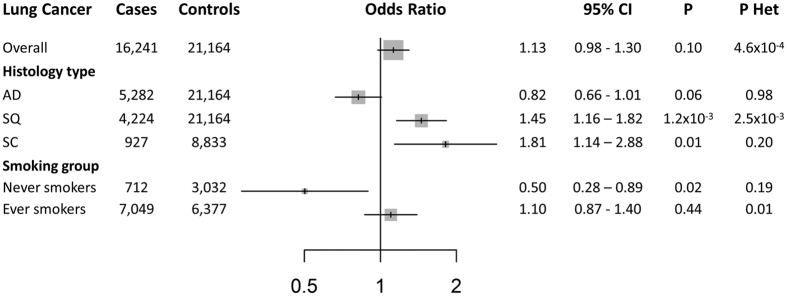
Forest plot of lung cancer risk for an increase of 1 SD of BMI (approximately 4.65 Kg/m^2^) observed in a likelihood-based MR approach. AD: Adenocarcinoma; SQ: squamous cell lung cancer; SC: small cell lung cancer; OR: Odds Ratio; 95%CI: 95% Confidence Interval; P: P value; P Het: P value of heterogeneity among individual SNP causal estimates.

The genetic instrument for BMI was positively associated with overall lung cancer risk: the estimated OR per genetically elevated one SD increase in BMI was 1.13 ([95%CI] = [0.98–1.30], P = 0.10). In analyses stratified by histology, the association between the BMI genetic instrument and risk was evident for SQ carcinoma (OR [95%CI] = 1.45 [1.16–1.62]; P = 1.19 × 10^−3^) and for SC carcinoma (OR [95%CI] = 1.81 [1.14–2.88]; P = 0.01), but not for AD (OR [95%CI] = 0.82 [0.66–1.01]; P = 0.06) (P value of heterogeneity among histological strata = 3 × 10^−5^) (Fig. 2). In the sample subset for which individual level data were available, smoking and sex stratified analysis were performed only for lung cancer overall due to the limited number of never smokers and the lack of power within histological types. The BMI instrument was inversely associated with overall lung cancer in never smokers (OR [95%CI] = 0.50 [0.28–0.89]; P = 0.02), while it remained positively correlated in ever smokers (OR [95%CI] = 1.10 [0.87–1.39]; P = 0.44) (P value of heterogeneity between never and ever smokers in overall = 0.01) (Fig. 2). In sex stratified analyses, no differences were observed between sex groups (P value of heterogeneity = 0.28). Meta-analysis results for the 97 SNPs (β_GD_) on the described phenotypes are presented in Supplementary Table S7.

### Sensitivity analyses

To evaluate the potential effect of pleiotropy on the causal effect estimates, several sensitivity analyses were performed. The first sensitivity analysis was the re-evaluation of the likelihood-based approach by removing the rs11030104 SNP (reported to be associated with smoking initiation), which did not notably alter the results (Supplementary Table S8). The weighted median analysis resulted in similar risk estimates, except for SC carcinoma which was attenuated (OR of 1.42 ([95%CI] = 0.66–3.06; P = 0.37)) (Supplementary Table S9). Finally, the analyses of the MR-Egger test did not detect directional pleiotropy effecting risk estimations (Supplementary Table S10).

## Discussion

This study aimed to evaluate whether Mendelian randomization can help to clarify the causal relationship between BMI and lung cancer risk. Large-scale GWAS initiatives provided an informative genetic instrument for BMI which was used in subsequent risk analysis in large numbers of lung cancer cases and control. Our results suggest that obesity may cause a higher risk of SQ and SC lung carcinoma. The absence of any association between our genetic instrument for BMI and smoking patterns suggests that these results are not explained by confounding by tobacco. These results are in stark contrast with most observational analyses indicating an inverse association between BMI and lung cancer risk.

Several assumptions are required for Mendelian randomization to provide consistent estimates of the causal effect of a putative risk factor on diseases, including a sufficiently strong association between the genetic instrument and exposure, and the absence of pleiotropy. Two main features of the Mendelian randomization methodologies we used to ensure that our results satisfy these assumptions or are robust to deviations from the assumptions. First of all, several genetic variants were interrogated as genetic instruments for the modifiable exposure at the same time, which reduces the probability of violating the conditions of the methodology regarding true association and pleiotropy-driven bias. Then, the two-sample Mendelian randomization approach^22^ allowed us to obtain the calibrated genetic effects from the largest existing genome-wide studies on obesity (GIANT consortium with 339,224 participants) and on lung cancer (TRICL, Epic and Axiom datasets with 16,572 cases and 21,480 controls).

The initial Mendelian randomization approach and subsequent sensitivity analyses showed a consistent risk effect of BMI for SQ and SC lung carcinoma subtypes. For the SQ subtype, a 1 SD increase in BMI (4.65 Kg/m^2^) conferred a 45% increased risk of lung cancer. In the case of SC carcinoma, the increase of risk was approximately 80%, higher than other histological subtypes. Despite the fact that our SC sample had power to detect a risk increase of 85%, the 1.81 risk increase detected is still sufficiently powered (77.4%) to consider this as a robust result. There was no evidence of any pleiotropic effects on the relative risk estimations. These results, together with the suggestive inverse effect for AD, could reflect different contributions of adiposity on each lung cancer subtype. Additionally, the analyses stratified by smoking status revealed an inverse association of genetically instrumented BMI in never smokers for overall lung cancer reducing the risk by as much as half. However, the analysis in never smokers could be slightly underpowered (72.4% of power to detect a risk of 2.0). Finally, a specific role of BMI regarding sex did not seem to be consistent.

Different hypotheses have been suggested as biological mechanisms for an association between obesity and cancer risk in general^25^. These include mechanisms involving sex hormone metabolism, insulin and insulin-like growth factor signaling, and adipokine pathophysiology^25^. In the case of lung cancer, sex hormone metabolism might not be influencing risk since no different effect have been observed regarding sex in this study, as well as other observational studies^3^. At the same time, a potential protective role of BMI on DNA damage from smoking or occupational exposures have been observed^6^. All of these elements point towards diverse and tissue-specific mechanisms rather than global systemic physiological explanation.

Our Mendelian randomization study does not support previous results from observational studies that obesity may decrease lung cancer risk overall. In contrast, our data indicate that obesity may cause an increased risk of SQ and SC lung carcinoma but not of AD.

## Additional Information

**How to cite this article**: Carreras-Torres, R. *et al*. The causal relevance of body mass index in different histological types of lung cancer: A Mendelian randomization study. *Sci. Rep.* **6**, 31121; doi: 10.1038/srep31121 (2016).

## Supplementary Material

Supplementary Information
