# Supplementary material for: The causal relevance of body mass index in different histological types of lung cancer: A Mendelian randomization study
Source: Sci Rep. 2016 Aug 4;6:31121. doi: 10.1038/srep31121 (PMC4973233; doi:10.1038/srep31121)

Original article:

The causal relevance of body mass index in different histological types of lung cancer. A Mendelian randomization study.

Robert Carreras-Torres[1], Philip C Haycock[2], Caroline L Relton[2], Richard M Martin[2,3], George Davey Smith[2], Peter Kraft[4], Chi Gao[4], Shelley Tworoger[4,5], Loïc Le Marchand[6], Lynne R Wilkens[6], Sungshim L Park[7], Christopher Haiman[7], John K Field[8], Michael Davies[8], Michael Marcus[8], Geoffrey Liu[9], Neil E Caporaso[10], David C Christiani[11], Yongyue Wei[11], Chu Chen[12], Jennifer A Doherty[13], Gianluca Severi[14], Gary E Goodman[15], Rayjean J Hung[16], Christopher I Amos[17], James McKay[1], Mattias Johansson[1], Paul Brennan[1]*.

[1] Section of Genetics, International Agency for Research on Cancer (IARC), Lyon, France.
[2] MRC Integrative Epidemiology Unit, School of Social and Community Medicine, University of Bristol, Bristol, UK.
[3] National Institute for Health Research Biomedical Research Unit in Nutrition, Diet and Lifestyle at University Hospitals Bristol NHS Foundation Trust and the University of Bristol, BS2 8AE, Bristol, UK.
[4] Department of Epidemiology, Harvard T.H. Chan School of Public Health, Boston, USA.
[5] Channing Division of Network Medicine, Brigham and Women's Hospital and Harvard Medical School, Boston, USA.
[6] Epidemiology Program, University of Hawaii Cancer Center, Honolulu, USA.
[7] Norris Comprehensive Cancer Center, Keck School of Medicine, University of Southern California, Los Angeles, USA.
[8] Roy Castle Lung Cancer Research Programme, The University of Liverpool Cancer Research Centre, Department of Molecular and Clinical Cancer Medicine, Institute of Translational Medicine, The University of Liverpool, Liverpool, UK.
[9] Ontario Cancer Institute, Princess Margaret Cancer Center, Toronto, ON, Canada.
[10] Genetic Epidemiology Branch, DCEG, National Cancer Institute, NIH, Rockville, USA.
[11] Departments of Environmental Health and Epidemiology, Harvard T. H. Chan School of Public Health and Department of Medicine, Massachusetts General Hospital/Harvard Medical School, Boston, USA.
[12] Program in Epidemiology, Division of Public Health Sciences, Fred Hutchinson Cancer Research Center, Seattle, USA.
[13] Department of Epidemiology, Geisel School of Medicine, Dartmouth College, Lebanon, USA.
[14] Human Genetics Foundation (HuGeF), Torino, Italy.

[15] Fred Hutchinson Cancer Research Center, Seattle, USA.
[16] Lunenfeld-Tanenbaum Research Institute of Mount Sinai Hospital, Toronto, Canada.
[17] Department of Biomedical Data Science, Geisel School of medicine, Dartmouth College, Lebanon, USA.

Running Head: Causality appraisal of body-mass index on lung cancer risk

Keywords: Body mass index, lung cancer, two-sample Mendelian randomization

***Corresponding author:**

Dr Paul Brennan,
Genetic Epidemiology Group,
International Agency for Research on Cancer,
150 Cours Albert Thomas,
69008 Lyon, France.
E-mail: gep@iarc.fr

Word count:
Abstract: 195
Text: 2,265

Supplementary Table S1 - Number of controls, overall cases, adenocarcinoma (AD) samples, squamous cell (SQ) samples and small cell (SC) lung cancer cases from the different studies with sample and smoking habits description within study. The largest sample combination numbers are the result of the TRICL, Axiom-TRICL and EPIC combination for Controls, Cases, AD and SQ, while for SC is the result of the IARC-ILCCO, Axiom-TRICL and EPIC combination.

Data type	Study	Sample	Controls	Cases	AD	SQ	SC
**Summary data**	**TRICL**	**Total**	15,861	11,348	3,441	3,275	0
**Individual level data**	**IARC-ILCCO**	**Total**	3,825	2,554	521	916	395
		**% ever smokers**	0.66	0.94	0.86	0.97	0.97
		**% males**	0.67	0.75	0.64	0.87	0.70
		**CARET**	354	362	98	68	60
		**Central Europe**	2,137	1,604	357	695	243
		**HUNT2/Tromso**	367	353	66	68	48
		**Estonia**	823	106	0	0	0
		**France**	144	129	0	85	44
	**Axiom-TRICL**	**Total**	3,881	3,597	1,403	690	288
		**% ever smokers**	0.68	0.89	0.82	0.96	0.98
		**% males**	0.51	0.52	0.43	0.80	0.52
		**CARET**	701	255	58	44	50
		**LCS**	492	517	263	23	63
		**NHS-HPFS**	708	615	0	0	0
		**LLP**	366	364	147	159	45
		**MCCS**	933	1,115	658	217	83
		**MEC**	234	243	96	32	29
		**Rusia**	447	488	181	215	18
	**EPIC**	**Total**	1,465	1,452	508	292	221
		**% ever smokers**	0.65	0.89	0.82	0.96	0.98
		**% males**	0.60	0.60	0.51	0.73	0.64
**Largest sample combination**		**Total**	**21,207**	**16,397**	**5,352**	**4,257**	**904**

Supplementary Table S2 - TRICL study rs number, chromosome and base position in hg19, genotyping and imputation information, and association parameters for analyzed BMI SNPs

									Overall			Adenocarcinoma			Squamous Cell		
**BMI SNP**	**CHR**	**BP**	**EA**	**OA**	**info_s1**	**RSQ_s2**	**RSQ_s3**	**info_s4**	**Effect**	**SE**	**P**	**Effect**	**SE**	**P**	**Effect**	**SE**	**P**
rs977747	1	47684677	T	G	0.99	0.78	0.98	1.00	-0.04	0.02	0.03	-0.03	0.03	0.24	-0.05	0.03	0.11
rs657452	1	49589847	A	G	1.00	1.00	1.00	1.00	0.00	0.02	0.96	0.01	0.03	0.74	0.02	0.03	0.57
rs11583200	1	50559820	C	T	1.00	0.96	1.00	1.00	0.00	0.02	0.99	0.01	0.03	0.69	-0.01	0.03	0.64
rs3101336	1	72751185	C	T	1.00	1.00	1.00	1.00	-0.02	0.02	0.33	-0.04	0.03	0.23	-0.03	0.03	0.30
rs12401738	1	78446761	A	G	1.00	0.98	0.98	1.00	0.05	0.02	0.01	0.02	0.03	0.50	0.10	0.03	0.00
rs17024393	1	110154688	C	T	0.99	0.97	0.99	0.99	-0.03	0.05	0.56	0.03	0.08	0.71	0.00	0.07	0.96
rs543874	1	177889480	G	A	1.00	0.98	0.97	1.00	-0.02	0.02	0.41	-0.01	0.04	0.76	-0.06	0.04	0.12
rs2820292	1	201784287	C	A	0.99	0.97	0.96	0.99	0.01	0.02	0.43	0.02	0.03	0.39	0.00	0.03	0.90
rs13021737	2	632348	G	A	0.99	0.98	0.98	0.99	0.02	0.02	0.35	-0.03	0.04	0.34	0.04	0.04	0.29
rs10182181	2	25150296	G	A	1.00	1.00	1.00	1.00	0.03	0.02	0.10	0.03	0.03	0.34	0.06	0.03	0.04
rs11126666	2	26928811	A	G	1.00	1.00	1.00	1.00	0.00	0.02	0.84	-0.07	0.03	0.02	0.02	0.03	0.58
rs1016287	2	59305625	T	C	0.99	0.98	0.98	0.99	-0.03	0.02	0.15	-0.05	0.03	0.12	-0.03	0.03	0.31
rs11688816	2	63053048	G	A	1.00	1.00	1.00	1.00	0.00	0.02	0.79	-0.01	0.03	0.66	-0.01	0.03	0.67
rs2121279	2	143043285	T	C	0.98	0.97	0.97	0.98	0.02	0.03	0.40	-0.08	0.04	0.05	0.05	0.04	0.22
rs1460676	2	164567689	C	T	1.00	0.99	0.99	1.00	0.02	0.02	0.50	0.04	0.04	0.26	0.01	0.04	0.73
rs1528435	2	181550962	T	C	0.99	0.98	0.99	0.99	0.00	0.02	0.79	-0.01	0.03	0.84	0.00	0.03	0.88
rs17203016	2	208255518	G	A	1.00	0.91	0.92	1.00	0.01	0.02	0.57	0.02	0.04	0.59	0.00	0.04	0.99
rs7599312	2	213413231	G	A	1.00	0.99	1.00	1.00	0.01	0.02	0.78	0.02	0.03	0.53	0.03	0.03	0.40
rs492400	2	219349752	C	T	1.00	0.76	0.87	1.00	-0.05	0.02	0.01	-0.02	0.03	0.50	-0.07	0.03	0.01
rs2176040	2	227092802	A	G	1.00	1.00	0.99	1.00	-0.02	0.02	0.39	-0.01	0.03	0.67	-0.03	0.03	0.28
rs6804842	3	25106437	G	A	1.00	0.87	0.86	1.00	-0.03	0.02	0.18	-0.04	0.03	0.18	-0.02	0.03	0.57
rs2365389	3	61236462	C	T	0.99	0.98	0.98	0.99	-0.02	0.02	0.20	-0.01	0.03	0.77	-0.01	0.03	0.74
rs3849570	3	81792112	A	C	1.00	0.89	0.93	0.99	0.00	0.02	0.93	-0.02	0.03	0.55	0.03	0.03	0.37
rs13078960	3	85807590	G	T	1.00	0.97	0.97	1.00	0.00	0.02	0.88	0.02	0.04	0.50	-0.03	0.04	0.42
rs16851483	3	141275436	T	G	1.00	0.99	0.99	1.00	0.01	0.04	0.73	-0.01	0.06	0.89	0.04	0.06	0.52
rs1516725	3	185824004	C	T	1.00	0.98	0.99	1.00	-0.03	0.03	0.33	-0.02	0.04	0.61	-0.03	0.04	0.54
rs10938397	4	45182527	G	A	1.00	0.87	0.89	1.00	-0.01	0.02	0.43	-0.06	0.03	0.05	-0.01	0.03	0.71
rs17001654	4	77129568	G	C	0.93	0.83	0.89	0.92	0.04	0.03	0.10	0.07	0.04	0.10	0.06	0.04	0.15
rs13107325	4	103188709	T	C	0.86	0.98	0.97	1.00	0.05	0.04	0.19	0.02	0.05	0.65	0.01	0.06	0.80
rs11727676	4	145659064	T	C	0.74	0.46	0.56	0.74	0.03	0.04	0.41	0.02	0.06	0.76	0.08	0.06	0.19
rs2112347	5	75015242	T	G	0.99	0.92	0.94	0.99	0.01	0.02	0.66	-0.02	0.03	0.46	0.00	0.03	0.97
rs7715256	5	153537893	G	T	1.00	1.00	1.00	1.00	-0.03	0.02	0.15	-0.05	0.03	0.07	-0.01	0.03	0.61
rs205262	6	34563164	G	A	1.00	1.00	1.00	1.00	0.01	0.02	0.54	0.00	0.03	0.93	0.02	0.03	0.45
rs2033529	6	40348653	G	A	1.00	1.00	1.00	1.00	-0.03	0.02	0.18	-0.02	0.03	0.44	-0.03	0.03	0.31
rs2207139	6	50845490	G	A	1.00	0.99	0.99	1.00	0.01	0.02	0.69	-0.04	0.04	0.27	0.05	0.04	0.22
rs9400239	6	108977663	C	T	0.99	0.98	0.99	0.99	-0.01	0.02	0.62	0.02	0.03	0.46	-0.04	0.03	0.14
rs9374842	6	120185665	T	C	1.00	0.99	0.99	1.00	0.01	0.02	0.53	0.01	0.03	0.82	0.05	0.03	0.14
rs13201877	6	137675541	G	A	0.98	0.93	0.93	0.98	-0.01	0.03	0.70	-0.02	0.04	0.67	0.03	0.04	0.43
rs13191362	6	163033350	A	G	1.00	0.99	1.00	1.00	0.04	0.03	0.22	0.03	0.04	0.52	0.00	0.05	0.95
rs1167827	7	75163169	G	A	1.00	1.00	1.00	0.95	-0.01	0.02	0.58	-0.02	0.03	0.44	0.00	0.03	0.87
rs6465468	7	95169514	T	G	0.97	0.92	0.94	0.97	0.01	0.02	0.52	0.00	0.03	1.00	0.03	0.03	0.41
rs17405819	8	76806584	T	C	1.00	1.00	1.00	0.99	-0.01	0.02	0.78	0.04	0.03	0.21	-0.02	0.03	0.52
rs16907751	8	81375457	C	T	0.98	0.95	0.96	0.98	-0.06	0.03	0.05	-0.05	0.05	0.32	-0.08	0.05	0.08
rs2033732	8	85079709	C	T	0.97	0.88	0.93	0.97	-0.02	0.02	0.44	0.00	0.03	0.94	-0.02	0.03	0.65
rs4740619	9	15634326	T	C	1.00	0.95	0.96	1.00	0.02	0.02	0.32	0.00	0.03	0.88	0.03	0.03	0.30
rs10968576	9	28414339	G	A	1.00	0.99	1.00	1.00	0.00	0.02	0.81	-0.02	0.03	0.45	0.05	0.03	0.07
rs6477694	9	111932342	C	T	1.00	1.00	1.00	1.00	-0.01	0.02	0.47	-0.02	0.03	0.40	0.01	0.03	0.82
rs1928295	9	120378483	T	C	1.00	1.00	1.00	1.00	0.01	0.02	0.61	0.02	0.03	0.52	0.00	0.03	0.98
rs10733682	9	129460914	A	G	1.00	0.99	0.99	1.00	-0.01	0.02	0.77	-0.03	0.03	0.21	0.02	0.03	0.43
rs7899106	10	87410904	G	A	0.99	0.98	0.97	0.99	-0.12	0.04	0.01	-0.14	0.07	0.05	-0.03	0.07	0.70
rs17094222	10	102395440	C	T	0.98	0.95	0.97	0.98	0.02	0.02	0.30	0.03	0.03	0.40	0.01	0.04	0.85
rs11191560	10	104869038	C	T	0.99	1.00	0.99	0.99	0.03	0.03	0.42	0.00	0.05	0.93	0.07	0.05	0.15
rs7903146	10	114758349	C	T	1.00	1.00	1.00	1.00	0.03	0.02	0.09	0.01	0.03	0.64	0.02	0.03	0.44
rs4256980	11	8673939	G	C	1.00	1.00	1.00	1.00	0.00	0.02	0.85	0.05	0.03	0.12	-0.02	0.03	0.54
rs11030104	11	27684517	A	G	1.00	1.00	1.00	1.00	0.04	0.02	0.11	0.00	0.03	0.91	0.08	0.03	0.02
rs2176598	11	43864278	T	C	0.99	1.00	1.00	0.99	0.00	0.02	0.94	-0.02	0.03	0.59	0.00	0.03	0.92
rs3817334	11	47650993	T	C	0.99	0.98	0.99	0.99	0.01	0.02	0.65	-0.02	0.03	0.40	0.03	0.03	0.36
rs12286929	11	115022404	G	A	0.99	0.96	0.98	0.99	0.05	0.02	0.00	0.07	0.03	0.01	0.04	0.03	0.16
rs7138803	12	50247468	A	G	1.00	1.00	1.00	1.00	-0.03	0.02	0.08	0.01	0.03	0.84	-0.08	0.03	0.01
rs11057405	12	122781897	G	A	1.00	0.99	0.99	1.00	-0.02	0.03	0.61	0.00	0.05	0.99	-0.01	0.05	0.81
rs12429545	13	54102206	A	G	1.00	1.00	1.00	1.00	0.03	0.03	0.27	0.01	0.04	0.74	0.10	0.04	0.02
rs9540493	13	66205704	A	G	0.98	0.75	0.98	0.97	0.00	0.02	0.88	0.03	0.03	0.38	0.00	0.03	0.91
rs1441264	13	79580919	A	G	1.00	1.00	1.00	1.00	0.02	0.02	0.25	0.02	0.03	0.39	0.00	0.03	0.97
rs10132280	14	25928179	C	A	0.97	0.92	0.94	0.97	0.02	0.02	0.44	-0.02	0.03	0.45	-0.03	0.03	0.32
rs12885454	14	29736838	C	A	0.99	0.93	0.93	0.99	0.04	0.02	0.02	0.04	0.03	0.23	0.03	0.03	0.32
rs11847697	14	30515112	T	C	0.94	0.96	0.97	0.96	0.07	0.05	0.14	0.01	0.07	0.94	0.02	0.08	0.83
rs7141420	14	79899454	T	C	1.00	0.98	0.99	1.00	-0.01	0.02	0.42	0.01	0.03	0.80	-0.03	0.03	0.29
rs3736485	15	51748610	A	G	0.98	0.97	0.97	0.97	0.03	0.02	0.16	0.01	0.03	0.69	0.05	0.03	0.07
rs16951275	15	68077168	T	C	1.00	0.99	1.00	1.00	0.03	0.02	0.14	0.01	0.03	0.77	0.05	0.03	0.14
rs7164727	15	73093991	T	C	1.00	0.94	0.96	1.00	0.03	0.02	0.11	0.01	0.03	0.76	0.05	0.03	0.13
rs758747	16	3627358	T	C	1.00	0.98	0.98	1.00	0.04	0.02	0.04	0.04	0.03	0.19	0.01	0.03	0.70
rs12446632	16	19935389	G	A	1.00	1.00	1.00	1.00	0.00	0.03	0.97	0.01	0.04	0.81	0.07	0.04	0.10
rs2650492	16	28333411	A	G	1.00	0.99	1.00	1.00	0.04	0.02	0.04	0.00	0.03	0.99	0.06	0.03	0.04
rs3888190	16	28889486	A	C	1.00	0.99	0.99	1.00	0.04	0.02	0.02	0.02	0.03	0.48	0.06	0.03	0.04
rs4787491	16	30015337	G	A	0.99	0.96	0.95	0.98	-0.02	0.02	0.20	-0.02	0.03	0.51	-0.01	0.03	0.75
rs9925964	16	31129895	A	G	1.00	1.00	1.00	1.00	0.03	0.02	0.16	-0.02	0.03	0.45	0.06	0.03	0.03
rs2080454	16	49062590	C	A	1.00	0.97	0.99	1.00	-0.02	0.02	0.36	-0.01	0.03	0.61	0.00	0.03	0.87
rs9914578	17	2005136	G	C	1.00	0.99	0.99	1.00	-0.04	0.02	0.06	-0.02	0.03	0.53	-0.06	0.03	0.10
rs1000940	17	5283252	G	A	1.00	1.00	1.00	1.00	-0.01	0.02	0.55	0.00	0.03	0.93	0.00	0.03	0.95
rs12940622	17	78615571	G	A	1.00	0.99	0.99	1.00	-0.02	0.02	0.25	-0.03	0.03	0.26	-0.03	0.03	0.28
rs1808579	18	21104888	C	T	1.00	1.00	1.00	1.00	0.03	0.02	0.07	0.01	0.03	0.64	0.05	0.03	0.05
rs7239883	18	40147671	G	A	1.00	1.00	1.00	1.00	-0.01	0.02	0.77	-0.01	0.03	0.68	0.01	0.03	0.73
rs7243357	18	56883319	T	G	0.98	0.93	0.95	0.99	0.01	0.02	0.75	0.01	0.04	0.77	0.02	0.04	0.62
rs6567160	18	57829135	C	T	1.00	0.97	0.98	1.00	0.03	0.02	0.15	0.04	0.03	0.27	0.03	0.03	0.40
rs17724992	19	18454825	A	G	1.00	0.88	0.93	1.00	0.03	0.02	0.19	0.02	0.03	0.60	0.06	0.03	0.07
rs29941	19	34309532	G	A	1.00	1.00	1.00	1.00	0.00	0.02	0.99	0.00	0.03	0.96	0.02	0.03	0.42
rs2075650	19	45395619	A	G	1.00	1.00	1.00	1.00	0.04	0.03	0.11	0.05	0.04	0.25	0.02	0.04	0.58
rs2287019	19	46202172	C	T	1.00	1.00	1.00	1.00	-0.01	0.02	0.66	-0.04	0.03	0.21	0.03	0.03	0.43
rs3810291	19	47569003	A	G	0.94	0.40	0.75	0.94	0.01	0.02	0.51	0.02	0.03	0.63	0.01	0.03	0.76
rs6091540	20	51087862	C	T	1.00	1.00	1.00	1.00	-0.01	0.02	0.47	0.03	0.03	0.42	-0.02	0.03	0.44
rs2836754	21	40291740	C	T	1.00	0.89	0.89	0.99	-0.02	0.02	0.40	0.00	0.03	0.88	-0.03	0.03	0.32

Supplementary Table S3 - IARC-ILCCO study rs number, chromosome and base position in hg19, genotyping and imputation information, and association parameters for analyzed BMI SNPs

								Overall									Adenocarcinoma			Squamous Cell			Small Cell		
								All sample			Never smokers			Ever smokers			All sample			All sample			All sample		
**SNP**	**CHR**	**BP**	**EA**	**OA**	**Freq1**	**Rsq**	**Genotyped**	**Effect**	**SE**	**P**	**Effect**	**SE**	**P**	**Effect**	**SE**	**P**	**Effect**	**SE**	**P**	**Effect**	**SE**	**P**	**Effect**	**SE**	**P**
rs977747	1	47684677	G	T	0.65	0.98	-	0.04	0.04	0.30	0.25	0.13	0.06	0.04	0.04	0.41	0.03	0.07	0.67	-0.03	0.06	0.61	0.04	0.08	0.66
rs657452	1	49589847	G	A	0.59	1.00	Genotyped	0.02	0.04	0.66	0.02	0.12	0.91	0.00	0.04	0.97	-0.01	0.07	0.95	0.02	0.06	0.73	0.05	0.08	0.53
rs1158320	1	50559820	T	C	0.60	1.00	Genotyped	0.00	0.04	0.99	-0.11	0.12	0.35	0.01	0.04	0.81	-0.05	0.07	0.45	0.03	0.06	0.61	-0.02	0.08	0.80
rs3101336	1	72751185	C	T	0.69	1.00	Genotyped	0.01	0.04	0.81	0.13	0.13	0.33	-0.02	0.05	0.73	0.04	0.07	0.58	-0.01	0.06	0.87	-0.11	0.08	0.20
rs1256698	1	75002193	A	G	0.61	0.67	-	0.05	0.05	0.26	-0.01	0.15	0.93	0.08	0.05	0.11	0.05	0.08	0.55	0.11	0.07	0.09	-0.04	0.09	0.67
rs1240173	1	78446761	G	A	0.67	0.98	-	-0.09	0.04	0.03	-0.08	0.13	0.53	-0.10	0.04	0.03	-0.09	0.07	0.21	-0.06	0.06	0.29	-0.21	0.08	0.01
rs1116564	1	96924097	T	C	0.54	0.98	-	0.02	0.04	0.69	-0.02	0.12	0.88	0.01	0.04	0.89	0.02	0.07	0.81	-0.04	0.06	0.47	0.04	0.08	0.61
rs1702439	1	110154688	T	C	0.94	0.98	-	0.03	0.09	0.75	-0.01	0.28	0.97	0.07	0.10	0.48	0.02	0.16	0.92	0.03	0.12	0.80	0.09	0.19	0.65
rs543874	1	177889480	A	G	0.85	0.99	-	-0.11	0.05	0.03	-0.28	0.16	0.09	-0.10	0.06	0.07	-0.11	0.09	0.23	0.01	0.07	0.86	-0.09	0.10	0.39
rs2820292	1	201784287	C	A	0.53	0.96	-	0.00	0.04	0.97	0.02	0.12	0.89	-0.04	0.04	0.40	0.04	0.07	0.53	-0.01	0.06	0.82	0.06	0.08	0.44
rs1302173	2	632348	G	A	0.82	0.99	-	0.02	0.05	0.73	0.15	0.17	0.37	-0.02	0.06	0.70	0.00	0.09	0.98	0.01	0.07	0.88	-0.02	0.10	0.85
rs1018218	2	25150296	A	G	0.57	1.00	Genotyped	-0.03	0.04	0.39	-0.02	0.13	0.89	-0.03	0.04	0.49	-0.04	0.07	0.58	-0.09	0.05	0.10	0.03	0.08	0.75
rs1112666	2	26928811	G	A	0.68	1.00	Genotyped	-0.02	0.04	0.57	-0.11	0.13	0.41	-0.03	0.05	0.53	0.07	0.08	0.38	-0.05	0.06	0.36	0.10	0.09	0.26
rs1168881	2	63053048	A	G	0.50	0.99	Genotyped	0.11	0.04	0.00	0.22	0.13	0.08	0.09	0.04	0.02	0.04	0.07	0.61	0.16	0.05	0.00	0.05	0.08	0.54
rs2121279	2	143043285	C	T	0.85	0.95	-	-0.06	0.05	0.24	-0.04	0.18	0.83	-0.07	0.06	0.27	0.08	0.10	0.42	-0.06	0.08	0.47	-0.18	0.11	0.10
rs1460676	2	164567689	T	C	0.81	0.97	-	-0.08	0.05	0.10	0.01	0.16	0.96	-0.07	0.05	0.21	0.02	0.09	0.83	-0.09	0.07	0.21	-0.15	0.10	0.12
rs1528435	2	181550962	T	C	0.64	0.97	-	-0.04	0.04	0.33	0.13	0.13	0.30	-0.07	0.04	0.14	-0.04	0.07	0.62	-0.11	0.06	0.06	0.03	0.08	0.72
rs1720301	2	208255518	A	G	0.81	0.83	-	0.01	0.05	0.90	0.09	0.18	0.61	0.04	0.06	0.54	-0.03	0.10	0.80	0.06	0.08	0.43	0.02	0.11	0.85
rs7599312	2	213413231	G	A	0.71	1.00	Genotyped	0.06	0.04	0.16	0.02	0.13	0.88	0.06	0.05	0.18	0.08	0.08	0.30	0.09	0.06	0.13	0.12	0.09	0.17
rs492400	2	219349752	T	C	0.59	0.89	-	0.04	0.04	0.32	-0.06	0.14	0.64	0.05	0.05	0.29	0.04	0.08	0.61	0.10	0.06	0.08	0.03	0.08	0.74
rs2176040	2	227092802	G	A	0.63	1.00	-	0.02	0.04	0.56	-0.17	0.13	0.18	0.03	0.04	0.49	0.08	0.07	0.26	0.02	0.06	0.78	0.04	0.08	0.66
rs6804842	3	25106437	G	A	0.61	0.81	-	0.01	0.04	0.78	0.02	0.14	0.87	0.01	0.05	0.85	0.01	0.08	0.93	0.05	0.06	0.43	0.07	0.09	0.41
rs2365389	3	61236462	C	T	0.60	0.95	-	-0.04	0.04	0.29	0.06	0.13	0.66	-0.05	0.04	0.25	-0.07	0.07	0.36	-0.04	0.06	0.45	0.02	0.08	0.80
rs3849570	3	81792112	C	A	0.67	0.98	-	-0.04	0.04	0.29	-0.01	0.13	0.94	-0.04	0.04	0.33	-0.05	0.07	0.50	-0.08	0.06	0.18	-0.15	0.08	0.07
rs1307896	3	85807590	T	G	0.83	0.97	-	-0.01	0.05	0.85	0.27	0.17	0.13	-0.04	0.06	0.44	0.10	0.09	0.27	-0.10	0.07	0.15	-0.13	0.10	0.20
rs1685148	3	141275436	G	T	0.93	0.99	-	0.06	0.08	0.47	0.18	0.25	0.48	0.06	0.08	0.45	0.07	0.14	0.62	0.13	0.11	0.23	0.06	0.16	0.71
rs1516725	3	185824004	C	T	0.88	0.99	-	-0.02	0.06	0.75	-0.07	0.18	0.69	-0.04	0.06	0.59	0.10	0.11	0.36	-0.02	0.08	0.85	0.00	0.12	0.97
rs1093839	4	45182527	A	G	0.57	0.84	-	-0.05	0.04	0.22	-0.08	0.14	0.55	-0.06	0.05	0.22	-0.05	0.08	0.50	0.03	0.06	0.64	-0.26	0.08	0.00
rs1700165	4	77129568	C	G	0.85	0.90	-	0.00	0.06	0.96	0.15	0.18	0.41	-0.06	0.06	0.38	0.05	0.10	0.60	-0.10	0.08	0.22	0.01	0.11	0.90
rs1310732	4	103188709	C	T	0.92	0.98	Genotyped	-0.07	0.07	0.34	-0.04	0.24	0.88	-0.05	0.08	0.50	-0.14	0.12	0.26	-0.05	0.10	0.65	-0.03	0.14	0.82
rs1172767	4	145659064	T	C	0.93	0.37	-	0.30	0.12	0.01	0.53	0.40	0.19	0.31	0.14	0.02	0.07	0.22	0.73	0.47	0.18	0.01	0.38	0.26	0.15
rs2112347	5	75015242	T	G	0.70	0.63	-	-0.02	0.05	0.76	-0.13	0.17	0.43	-0.02	0.06	0.77	-0.12	0.10	0.22	-0.02	0.08	0.78	-0.05	0.11	0.66
rs7715256	5	153537893	T	G	0.60	0.99	-	0.02	0.04	0.56	-0.02	0.12	0.86	0.01	0.04	0.84	0.02	0.07	0.74	0.02	0.06	0.70	-0.09	0.08	0.24
rs205262	6	34563164	A	G	0.75	1.00	Genotyped	-0.01	0.04	0.81	0.05	0.14	0.73	-0.01	0.05	0.90	-0.04	0.08	0.64	-0.10	0.06	0.10	0.03	0.09	0.72
rs2033529	6	40348653	A	G	0.69	1.00	Genotyped	0.02	0.04	0.69	-0.17	0.13	0.19	0.05	0.05	0.29	-0.01	0.07	0.94	-0.01	0.06	0.88	0.12	0.09	0.15
rs2207139	6	50845490	A	G	0.77	0.99	-	-0.02	0.05	0.62	-0.22	0.14	0.13	0.01	0.05	0.84	-0.02	0.09	0.78	-0.01	0.07	0.88	0.03	0.10	0.79
rs9400239	6	108977663	C	T	0.65	0.96	-	-0.05	0.04	0.21	-0.04	0.13	0.79	-0.07	0.05	0.12	-0.01	0.08	0.92	-0.09	0.06	0.14	0.03	0.09	0.74
rs9374842	6	120185665	T	C	0.72	0.99	-	0.00	0.04	1.00	-0.07	0.14	0.60	-0.01	0.05	0.92	0.00	0.08	0.98	0.01	0.06	0.90	-0.13	0.09	0.15
rs1320187	6	137675541	A	G	0.85	0.88	-	0.04	0.06	0.52	0.02	0.18	0.91	0.01	0.06	0.84	-0.03	0.11	0.75	0.05	0.09	0.59	0.13	0.12	0.28
rs1319136	6	163033350	A	G	0.91	0.99	-	0.04	0.06	0.51	-0.27	0.20	0.18	0.08	0.07	0.26	-0.06	0.11	0.60	0.03	0.09	0.73	0.02	0.13	0.89
rs1167827	7	75163169	G	A	0.54	1.00	Genotyped	-0.06	0.04	0.11	0.04	0.12	0.73	-0.06	0.04	0.16	-0.04	0.07	0.59	-0.05	0.06	0.41	-0.15	0.08	0.06
rs2245368	7	76608143	T	C	0.73	0.00	-	-5.06	2.24	0.02	2.94	7.19	0.68	-5.03	2.48	0.04	-7.95	4.10	0.05	-0.54	3.25	0.87	-6.44	4.61	0.16
rs9641123	7	93197732	G	C	0.58	0.96	-	-0.01	0.04	0.72	0.06	0.13	0.65	-0.04	0.04	0.41	-0.13	0.07	0.07	0.02	0.06	0.77	0.07	0.08	0.36
rs6465468	7	95169514	G	T	0.72	0.86	-	0.02	0.04	0.65	-0.10	0.14	0.46	0.04	0.05	0.38	-0.06	0.08	0.43	0.05	0.06	0.44	-0.02	0.09	0.79
rs1740581	8	76806584	T	C	0.68	1.00	-	-0.09	0.04	0.03	-0.10	0.13	0.45	-0.06	0.04	0.14	0.04	0.07	0.63	-0.04	0.06	0.48	-0.04	0.08	0.65
rs1690775	8	81375457	C	T	0.87	0.96	-	0.00	0.06	0.96	0.21	0.20	0.29	-0.04	0.07	0.57	-0.07	0.11	0.54	0.03	0.09	0.78	-0.15	0.12	0.21
rs2033732	8	85079709	C	T	0.73	0.86	-	-0.02	0.05	0.61	-0.21	0.15	0.16	0.00	0.05	0.99	0.06	0.09	0.49	-0.09	0.07	0.19	0.04	0.10	0.66
rs4740619	9	15634326	T	C	0.58	0.92	-	0.03	0.04	0.49	-0.13	0.13	0.32	0.05	0.04	0.23	0.02	0.07	0.78	0.07	0.06	0.24	0.01	0.08	0.92
rs1096857	9	28414339	A	G	0.67	1.00	-	0.03	0.04	0.42	0.05	0.13	0.70	0.03	0.04	0.51	0.08	0.07	0.27	-0.06	0.06	0.31	0.14	0.08	0.10
rs6477694	9	111932342	T	C	0.61	1.00	Genotyped	0.02	0.04	0.57	0.06	0.13	0.62	0.01	0.04	0.89	0.02	0.07	0.83	-0.03	0.06	0.60	-0.02	0.08	0.78
rs1928295	9	120378483	T	C	0.53	1.00	Genotyped	-0.06	0.04	0.13	0.06	0.12	0.61	-0.06	0.04	0.14	-0.11	0.07	0.11	-0.07	0.05	0.12	-0.09	0.08	0.24
rs1073368	9	129460914	A	G	0.50	0.99	Genotyped	-0.03	0.04	0.41	-0.17	0.12	0.18	-0.01	0.04	0.85	-0.01	0.07	0.87	-0.06	0.05	0.26	-0.10	0.08	0.21
rs7899106	10	87410904	A	G	0.97	0.88	-	0.30	0.11	0.01	0.04	0.37	0.91	0.38	0.12	0.00	0.46	0.22	0.04	0.20	0.16	0.22	0.03	0.21	0.87
rs1709422	10	102395440	T	C	0.81	0.95	-	0.05	0.05	0.34	0.10	0.16	0.52	0.03	0.05	0.58	0.05	0.09	0.57	0.17	0.07	0.02	-0.02	0.10	0.88
rs1119156	10	104869038	T	C	0.90	1.00	-	0.06	0.06	0.32	0.16	0.22	0.46	0.08	0.07	0.29	0.03	0.12	0.78	0.04	0.09	0.65	0.06	0.14	0.65
rs7903146	10	114758349	C	T	0.75	1.00	Genotyped	0.05	0.04	0.21	-0.07	0.14	0.64	0.07	0.05	0.17	0.06	0.08	0.42	0.01	0.06	0.93	0.16	0.09	0.07
rs4256980	11	8673939	G	C	0.60	0.97	-	0.01	0.04	0.71	0.07	0.13	0.57	0.02	0.04	0.63	0.04	0.07	0.57	0.08	0.06	0.15	0.00	0.08	0.99
rs1103010	11	27684517	A	G	0.80	1.00	Genotyped	-0.02	0.05	0.71	-0.04	0.15	0.78	-0.05	0.05	0.36	0.03	0.09	0.77	-0.01	0.07	0.92	-0.12	0.10	0.20
rs2176598	11	43864278	C	T	0.76	1.00	-	0.03	0.04	0.56	0.12	0.15	0.41	0.03	0.05	0.59	-0.04	0.08	0.67	0.04	0.06	0.49	0.10	0.09	0.27
rs3817334	11	47650993	C	T	0.57	0.97	-	0.02	0.04	0.66	0.09	0.13	0.48	0.00	0.04	0.97	0.10	0.07	0.18	-0.01	0.06	0.83	0.04	0.08	0.61
rs1228692	11	115022404	A	G	0.43	0.97	-	-0.03	0.04	0.45	0.07	0.13	0.60	-0.06	0.04	0.17	0.00	0.07	0.99	-0.08	0.06	0.15	0.00	0.08	1.00
rs7138803	12	50247468	G	A	0.55	1.00	Genotyped	0.00	0.04	0.93	-0.17	0.12	0.17	0.01	0.04	0.83	-0.15	0.07	0.03	0.05	0.06	0.41	0.02	0.08	0.78
rs1105740	12	122781897	G	A	0.88	0.93	Genotyped	0.02	0.06	0.74	-0.05	0.19	0.80	0.03	0.07	0.61	0.09	0.12	0.41	0.02	0.09	0.78	0.04	0.13	0.75
rs1242954	13	54102206	G	A	0.89	1.00	Genotyped	-0.14	0.06	0.02	-0.17	0.18	0.34	-0.15	0.07	0.02	-0.12	0.11	0.24	-0.19	0.08	0.02	0.06	0.13	0.62
rs9540493	13	66205704	G	A	0.60	0.99	-	0.03	0.04	0.51	0.18	0.13	0.15	0.02	0.04	0.61	0.00	0.07	1.00	0.02	0.06	0.74	0.02	0.08	0.85
rs1441264	13	79580919	A	G	0.60	1.00	Genotyped	0.03	0.04	0.39	0.21	0.13	0.11	0.01	0.04	0.75	0.12	0.07	0.10	-0.05	0.06	0.35	0.08	0.08	0.30
rs1013228	14	25928179	C	A	0.72	0.87	-	-0.04	0.05	0.42	-0.06	0.15	0.67	-0.03	0.05	0.52	-0.08	0.08	0.35	-0.10	0.07	0.14	0.02	0.10	0.82
rs1288545	14	29736838	C	A	0.68	0.92	-	0.11	0.04	0.01	0.23	0.14	0.09	0.10	0.05	0.04	0.15	0.08	0.05	0.07	0.06	0.25	0.08	0.09	0.35
rs1184769	14	30515112	C	T	0.97	0.95	-	-0.23	0.10	0.02	-0.22	0.32	0.50	-0.26	0.12	0.02	-0.09	0.18	0.64	-0.07	0.15	0.66	-0.56	0.18	0.00
rs7141420	14	79899454	T	C	0.57	0.97	-	-0.01	0.04	0.75	0.00	0.12	0.99	-0.01	0.04	0.82	0.03	0.07	0.66	-0.04	0.05	0.47	0.14	0.08	0.07
rs3736485	15	51748610	G	A	0.63	0.95	-	-0.05	0.04	0.20	0.06	0.13	0.66	-0.09	0.04	0.04	-0.09	0.07	0.19	-0.02	0.06	0.76	0.00	0.08	0.97
rs1695127	15	68077168	T	C	0.80	0.99	-	0.03	0.05	0.55	-0.22	0.15	0.16	0.04	0.05	0.50	-0.02	0.09	0.79	0.04	0.07	0.57	0.10	0.10	0.31
rs7164727	15	73093991	T	C	0.65	0.94	-	0.04	0.04	0.38	0.14	0.14	0.30	0.03	0.05	0.47	-0.03	0.07	0.65	0.03	0.06	0.66	0.07	0.09	0.42
rs758747	16	3627358	C	T	0.74	0.97	Genotyped	-0.03	0.04	0.44	-0.24	0.13	0.07	-0.03	0.05	0.56	-0.05	0.08	0.52	0.01	0.06	0.83	-0.08	0.09	0.37
rs1244663	16	19935389	G	A	0.85	1.00	Genotyped	-0.01	0.05	0.92	0.02	0.18	0.90	-0.02	0.06	0.78	0.17	0.10	0.09	0.04	0.08	0.56	-0.18	0.10	0.08
rs2650492	16	28333411	G	A	0.64	0.99	Genotyped	-0.02	0.04	0.62	0.10	0.13	0.46	-0.04	0.04	0.35	0.01	0.07	0.89	-0.06	0.06	0.31	0.05	0.08	0.53
rs3888190	16	28889486	C	A	0.58	0.99	-	-0.03	0.04	0.39	0.02	0.12	0.87	-0.04	0.04	0.35	-0.12	0.07	0.10	-0.05	0.05	0.33	-0.04	0.08	0.63
rs4787491	16	30015337	G	A	0.52	0.96	-	-0.05	0.04	0.18	0.07	0.12	0.54	-0.07	0.04	0.08	-0.05	0.07	0.52	-0.06	0.05	0.27	-0.12	0.08	0.12
rs9925964	16	31129895	A	G	0.63	1.00	-	0.03	0.04	0.41	-0.10	0.12	0.43	0.04	0.04	0.42	-0.07	0.07	0.30	0.03	0.06	0.55	0.09	0.08	0.25
rs2080454	16	49062590	A	C	0.59	0.96	-	0.01	0.04	0.88	-0.07	0.12	0.56	0.03	0.04	0.56	-0.13	0.07	0.07	0.01	0.06	0.86	0.06	0.08	0.49
rs1558902	16	53803574	T	A	0.55	0.90	-	0.01	0.04	0.75	0.19	0.13	0.15	-0.02	0.04	0.67	0.06	0.07	0.42	0.06	0.06	0.27	-0.06	0.08	0.44
rs9914578	17	2005136	C	G	0.81	0.93	-	0.08	0.05	0.11	0.16	0.16	0.33	0.05	0.05	0.36	-0.11	0.09	0.21	0.11	0.07	0.12	0.15	0.10	0.14
rs1000940	17	5283252	A	G	0.69	1.00	Genotyped	0.00	0.04	0.95	-0.04	0.13	0.78	0.00	0.05	1.00	0.02	0.07	0.77	-0.04	0.06	0.47	-0.09	0.08	0.29
rs1294062	17	78615571	G	A	0.57	0.98	-	0.08	0.04	0.05	0.09	0.12	0.50	0.08	0.04	0.07	0.04	0.07	0.53	0.11	0.06	0.05	0.14	0.08	0.08
rs1808579	18	21104888	C	T	0.52	1.00	Genotyped	0.00	0.04	0.95	0.04	0.12	0.78	-0.01	0.04	0.81	-0.11	0.07	0.12	0.00	0.06	0.94	0.12	0.08	0.13
rs7239883	18	40147671	A	G	0.57	1.00	Genotyped	-0.04	0.04	0.32	-0.04	0.13	0.75	-0.04	0.04	0.35	0.00	0.07	0.99	-0.04	0.05	0.41	0.00	0.08	0.97
rs7243357	18	56883319	T	G	0.83	0.93	-	-0.04	0.05	0.42	0.17	0.17	0.33	-0.05	0.06	0.40	-0.08	0.09	0.40	-0.02	0.07	0.80	-0.02	0.11	0.85
rs6567160	18	57829135	T	C	0.78	0.94	-	-0.05	0.05	0.27	0.00	0.15	1.00	-0.04	0.05	0.39	-0.12	0.08	0.14	-0.03	0.07	0.70	-0.06	0.09	0.50
rs1772499	19	18454825	A	G	0.73	0.83	-	0.01	0.05	0.88	0.12	0.16	0.43	-0.02	0.05	0.65	-0.05	0.08	0.58	0.08	0.07	0.25	-0.01	0.10	0.91
rs29941	19	34309532	G	A	0.68	1.00	Genotyped	-0.04	0.04	0.29	-0.02	0.13	0.88	-0.07	0.05	0.14	-0.09	0.07	0.24	0.02	0.06	0.71	-0.11	0.08	0.20
rs2075650	19	45395619	A	G	0.83	1.00	Genotyped	0.04	0.05	0.46	-0.17	0.16	0.29	0.07	0.06	0.22	0.06	0.10	0.56	0.03	0.07	0.72	0.09	0.11	0.40
rs2287019	19	46202172	C	T	0.77	0.99	Genotyped	-0.03	0.05	0.49	-0.06	0.14	0.69	-0.01	0.05	0.87	-0.07	0.08	0.39	0.00	0.07	0.98	0.02	0.10	0.84
rs3810291	19	47569003	A	G	0.66	0.79	-	0.00	0.05	0.94	0.09	0.14	0.55	-0.02	0.05	0.71	-0.07	0.08	0.41	0.03	0.07	0.61	0.02	0.09	0.80
rs6091540	20	51087862	C	T	0.75	0.98	-	0.03	0.04	0.47	0.05	0.14	0.74	0.04	0.05	0.45	0.15	0.08	0.05	0.02	0.06	0.77	0.03	0.09	0.76
rs2836754	21	40291740	C	T	0.59	0.88	-	0.03	0.04	0.44	0.04	0.13	0.75	0.03	0.05	0.57	-0.01	0.08	0.95	0.05	0.06	0.41	-0.01	0.09	0.88

Supplementary Table S4 - Axiom study rs number, chromosome and base position in hg19, genotyping and imputation information, and association parameters for analyzed BMI SNPs

								Overall									Adenocarcinoma			Squamous Cell			Small Cell		
								All sample			Never smokers			Ever smokers			All sample			All sample			All sample		
SNP	CHR	BP	EA	OA	Freq1	Info	Genotyped	Effect	SE	P	Effect	SE	P	Effect	SE	P	Effect	SE	P	Effect	SE	P	Effect	SE	P
rs977747	1	47684677	T	G	0.54	0.58	-	0.08	0.05	0.09	0.05	0.12	0.66	0.08	0.05	0.13	0.17	0.07	0.01	0.03	0.09	0.72	0.10	0.12	0.41
rs657452	1	49589847	A	G	0.57	0.81	-	-0.01	0.04	0.72	0.04	0.10	0.69	-0.05	0.05	0.23	0.00	0.05	0.96	0.11	0.07	0.11	-0.13	0.10	0.22
rs11583200	1	50559820	C	T	0.53	0.25	-	-0.14	0.07	0.04	-0.17	0.18	0.34	-0.11	0.08	0.16	-0.23	0.10	0.02	-0.05	0.13	0.73	0.11	0.19	0.55
rs3101336	1	72751185	T	C	0.66	1.00	-	0.05	0.04	0.14	0.03	0.09	0.78	0.05	0.04	0.26	0.09	0.05	0.09	0.12	0.07	0.07	-0.04	0.09	0.70
rs12566985	1	75002193	G	A	0.52	0.94	-	-0.05	0.04	0.16	-0.09	0.09	0.29	-0.03	0.04	0.46	-0.03	0.05	0.51	-0.09	0.07	0.15	0.12	0.09	0.19
rs12401738	1	78446761	G	A	0.28	0.65	-	-0.18	0.05	0.00	-0.05	0.12	0.66	-0.18	0.05	0.00	-0.08	0.07	0.23	-0.10	0.09	0.26	-0.22	0.12	0.07
rs11165643	1	96924097	C	T	0.58	0.98	-	0.02	0.03	0.65	0.01	0.09	0.92	0.03	0.04	0.52	-0.05	0.05	0.32	-0.06	0.06	0.39	-0.07	0.09	0.44
rs17024393	1	110154688	T	C	0.03	0.95	-	0.07	0.10	0.47	0.20	0.26	0.43	0.09	0.12	0.42	0.19	0.14	0.19	0.03	0.17	0.84	0.10	0.28	0.72
rs543874	1	177889480	A	G	0.19	1.00	Genotyped	-0.06	0.04	0.15	-0.05	0.11	0.63	-0.06	0.05	0.24	-0.15	0.06	0.01	-0.17	0.08	0.03	-0.03	0.11	0.77
rs2820292	1	201784287	A	C	0.48	0.71	-	0.06	0.04	0.13	0.16	0.10	0.11	0.04	0.05	0.36	0.08	0.06	0.18	0.10	0.07	0.16	0.11	0.11	0.30
rs13021737	2	632348	A	G	0.83	0.99	-	0.02	0.04	0.62	-0.04	0.11	0.72	0.04	0.05	0.42	0.05	0.06	0.43	0.05	0.08	0.55	-0.09	0.12	0.45
rs10182181	2	25150296	A	G	0.47	0.99	-	-0.02	0.03	0.48	0.00	0.09	0.98	-0.04	0.04	0.29	0.00	0.05	0.97	0.04	0.06	0.52	-0.09	0.09	0.30
rs11126666	2	26928811	G	A	0.30	0.85	-	-0.02	0.04	0.71	0.07	0.10	0.50	-0.03	0.05	0.50	-0.08	0.06	0.19	0.09	0.08	0.27	-0.11	0.11	0.33
rs1016287	2	59305625	T	C	0.66	0.74	-	-0.05	0.04	0.20	-0.06	0.11	0.55	-0.06	0.05	0.24	-0.12	0.06	0.04	-0.02	0.08	0.76	-0.08	0.11	0.46
rs11688816	2	63053048	G	A	0.45	0.87	-	-0.06	0.04	0.09	-0.12	0.09	0.21	-0.06	0.04	0.15	-0.10	0.05	0.04	-0.04	0.07	0.54	0.06	0.10	0.54
rs2121279	2	143043285	C	T	0.07	0.46	-	0.12	0.10	0.21	0.61	0.25	0.01	0.01	0.11	0.91	0.24	0.14	0.07	-0.02	0.17	0.93	0.09	0.26	0.73
rs1460676	2	164567689	T	C	0.19	0.47	-	-0.01	0.06	0.92	-0.23	0.16	0.17	0.05	0.07	0.48	0.01	0.09	0.93	-0.11	0.12	0.36	0.27	0.18	0.12
rs1528435	2	181550962	C	T	0.61	0.09	-	0.06	0.12	0.60	-0.13	0.29	0.65	0.06	0.14	0.65	0.13	0.17	0.44	0.23	0.22	0.30	0.14	0.32	0.65
rs17203016	2	208255518	A	G	0.16	0.08	-	0.07	0.17	0.67	-0.48	0.42	0.26	0.19	0.19	0.34	-0.16	0.23	0.48	0.88	0.33	0.01	-0.37	0.43	0.39
rs7599312	2	213413231	G	A	0.26	1.00	Genotyped	-0.02	0.04	0.55	-0.23	0.10	0.02	0.02	0.04	0.60	-0.02	0.05	0.74	0.02	0.07	0.80	0.09	0.10	0.41
rs492400	2	219349752	C	T	0.59	0.98	-	0.02	0.03	0.67	0.15	0.09	0.09	-0.02	0.04	0.61	0.05	0.05	0.27	-0.05	0.06	0.47	-0.11	0.09	0.22
rs2176040	2	227092802	A	G	0.66	1.00	-	-0.01	0.04	0.87	0.06	0.09	0.50	-0.04	0.04	0.39	0.02	0.05	0.69	0.01	0.07	0.84	-0.06	0.10	0.50
rs6804842	3	25106437	A	G	0.56	0.43	-	0.01	0.05	0.83	0.04	0.13	0.79	0.02	0.06	0.70	-0.02	0.07	0.79	-0.14	0.10	0.15	-0.15	0.14	0.30
rs2365389	3	61236462	C	T	0.56	0.21	-	0.05	0.07	0.47	-0.09	0.18	0.62	0.07	0.09	0.39	0.07	0.10	0.47	0.04	0.14	0.79	0.26	0.20	0.19
rs3849570	3	81792112	C	A	0.36	0.84	-	0.06	0.04	0.10	0.17	0.10	0.09	0.06	0.05	0.18	0.13	0.05	0.02	0.04	0.07	0.61	0.02	0.10	0.88
rs13078960	3	85807590	T	G	0.18	0.97	-	-0.01	0.04	0.74	-0.07	0.11	0.49	-0.02	0.05	0.70	0.01	0.06	0.93	-0.05	0.08	0.57	-0.06	0.11	0.63
rs16851483	3	141275436	G	T	0.09	0.98	-	0.13	0.07	0.04	0.16	0.16	0.32	0.12	0.08	0.14	0.20	0.09	0.03	0.11	0.12	0.35	0.46	0.20	0.02
rs1516725	3	185824004	T	C	0.87	0.70	-	-0.10	0.06	0.08	-0.08	0.15	0.62	-0.11	0.07	0.11	-0.10	0.08	0.22	-0.10	0.11	0.38	-0.13	0.16	0.42
rs10938397	4	45182527	A	G	0.42	1.00	Genotyped	0.02	0.03	0.58	0.05	0.09	0.60	0.01	0.04	0.89	0.06	0.05	0.24	0.04	0.06	0.51	0.10	0.09	0.26
rs17001654	4	77129568	C	G	0.15	0.87	-	0.00	0.05	0.93	0.07	0.13	0.61	-0.03	0.06	0.58	-0.02	0.07	0.78	0.08	0.10	0.44	-0.08	0.13	0.54
rs13107325	4	103188709	C	T	0.07	1.00	Genotyped	-0.08	0.06	0.18	-0.06	0.17	0.70	-0.04	0.07	0.57	-0.10	0.09	0.24	-0.25	0.11	0.02	-0.05	0.17	0.78
rs11727676	4	145659064	T	C	0.08	0.66	-	0.03	0.07	0.71	0.24	0.19	0.20	0.00	0.08	0.99	0.00	0.10	0.99	-0.13	0.13	0.34	0.33	0.22	0.13
rs2112347	5	75015242	T	G	0.38	0.99	Genotyped	0.00	0.04	0.97	-0.23	0.09	0.01	0.05	0.04	0.24	-0.03	0.05	0.61	-0.07	0.06	0.25	0.02	0.10	0.87
rs7715256	5	153537893	G	T	0.48	0.31	-	-0.09	0.06	0.15	-0.09	0.15	0.58	-0.11	0.07	0.11	-0.07	0.09	0.43	-0.15	0.11	0.18	-0.10	0.16	0.53
rs2033529	6	40348653	A	G	0.23	0.46	-	-0.09	0.06	0.11	-0.16	0.15	0.26	-0.10	0.07	0.17	-0.12	0.08	0.15	-0.05	0.11	0.63	-0.10	0.15	0.51
rs2207139	6	50845490	A	G	0.19	0.91	-	-0.08	0.05	0.08	-0.03	0.12	0.82	-0.05	0.05	0.31	-0.07	0.06	0.25	-0.11	0.08	0.19	-0.18	0.12	0.13
rs9400239	6	108977663	T	C	0.69	0.94	-	0.01	0.04	0.80	0.05	0.09	0.60	0.03	0.04	0.44	0.02	0.05	0.67	0.00	0.07	0.97	0.22	0.10	0.02
rs9374842	6	120185665	C	T	0.77	0.27	-	0.02	0.08	0.79	0.06	0.19	0.74	-0.04	0.09	0.65	0.11	0.11	0.31	-0.07	0.14	0.62	0.13	0.20	0.53
rs13201877	6	137675541	A	G	0.12	0.19	-	0.24	0.12	0.04	0.09	0.28	0.74	0.27	0.14	0.04	0.37	0.17	0.03	-0.13	0.20	0.52	0.22	0.31	0.49
rs13191362	6	163033350	A	G	0.08	0.04	-	-0.28	0.32	0.38	-0.42	0.80	0.59	-0.39	0.37	0.30	-1.05	0.44	0.02	-0.34	0.59	0.56	1.97	0.87	0.02
rs1167827	7	75163169	A	G	0.45	0.51	-	-0.04	0.05	0.35	0.10	0.12	0.42	-0.05	0.06	0.38	-0.07	0.07	0.31	0.02	0.09	0.84	-0.11	0.13	0.38
rs2245368	7	76608143	C	T	0.73	0.13	-	-0.26	0.11	0.02	-0.03	0.28	0.92	-0.34	0.13	0.01	-0.25	0.16	0.11	-0.39	0.21	0.07	-0.11	0.30	0.71
rs9641123	7	93197732	G	C	0.34	0.41	-	0.11	0.06	0.06	0.30	0.14	0.04	0.07	0.06	0.25	0.15	0.08	0.05	0.10	0.10	0.33	0.42	0.15	0.01
rs6465468	7	95169514	G	T	0.25	0.22	-	0.03	0.08	0.75	0.10	0.21	0.62	-0.03	0.10	0.77	0.14	0.12	0.24	0.12	0.16	0.44	0.07	0.22	0.74
rs17405819	8	76806584	T	C	0.30	0.67	-	-0.05	0.05	0.25	-0.14	0.12	0.23	-0.02	0.05	0.72	-0.09	0.06	0.14	0.02	0.08	0.77	0.01	0.12	0.97
rs16907751	8	81375457	C	T	0.11	0.99	-	0.04	0.05	0.44	-0.12	0.13	0.35	0.10	0.06	0.12	-0.05	0.08	0.47	-0.05	0.10	0.58	0.01	0.15	0.92
rs2033732	8	85079709	T	C	0.75	0.03	-	0.15	0.25	0.55	-0.26	0.62	0.68	0.12	0.29	0.69	0.28	0.35	0.42	-0.13	0.47	0.78	0.48	0.68	0.47
rs4740619	9	15634326	T	C	0.52	0.77	-	-0.01	0.04	0.87	-0.03	0.10	0.79	0.00	0.05	0.98	-0.06	0.05	0.23	0.05	0.07	0.52	-0.02	0.10	0.87
rs10968576	9	28414339	A	G	0.30	1.00	Genotyped	0.02	0.04	0.55	-0.15	0.09	0.11	0.06	0.04	0.14	0.02	0.05	0.70	0.11	0.07	0.10	-0.07	0.09	0.48
rs6477694	9	111932342	C	T	0.63	0.99	Genotyped	0.01	0.04	0.76	-0.05	0.09	0.56	0.04	0.04	0.39	0.05	0.05	0.35	0.16	0.07	0.02	-0.07	0.10	0.48
rs1928295	9	120378483	T	C	0.44	0.68	-	0.00	0.04	0.92	-0.18	0.11	0.09	0.03	0.05	0.53	-0.01	0.06	0.92	-0.03	0.08	0.70	0.10	0.11	0.38
rs10733682	9	129460914	A	G	0.44	0.12	-	-0.08	0.10	0.42	-0.30	0.25	0.23	-0.11	0.11	0.31	-0.18	0.14	0.18	-0.01	0.18	0.94	0.04	0.26	0.88
rs7899106	10	87410904	A	G	0.05	0.03	-	-0.65	0.43	0.13	-1.60	1.10	0.14	-0.14	0.49	0.78	-0.75	0.60	0.21	-0.92	0.78	0.24	0.39	1.16	0.73
rs17094222	10	102395440	T	C	0.24	0.35	-	-0.04	0.07	0.55	0.02	0.17	0.92	-0.05	0.08	0.53	0.00	0.09	0.98	-0.06	0.12	0.62	0.05	0.18	0.77
rs11191560	10	104869038	T	C	0.10	1.00	-	0.04	0.06	0.51	0.27	0.16	0.10	0.01	0.07	0.89	-0.05	0.08	0.54	0.02	0.11	0.85	-0.02	0.16	0.91
rs7903146	10	114758349	C	T	0.27	1.00	Genotyped	0.01	0.04	0.90	-0.12	0.10	0.21	0.04	0.04	0.30	-0.02	0.05	0.72	0.00	0.07	0.96	0.01	0.10	0.95
rs4256980	11	8673939	C	G	0.62	0.99	-	0.06	0.04	0.08	-0.08	0.09	0.37	0.11	0.04	0.01	0.00	0.05	0.94	0.10	0.06	0.11	0.27	0.09	0.00
rs11030104	11	27684517	A	G	0.22	0.99	-	0.08	0.04	0.07	0.18	0.11	0.09	0.05	0.05	0.29	-0.02	0.06	0.70	0.08	0.08	0.33	0.09	0.12	0.43
rs2176598	11	43864278	T	C	0.75	0.82	-	-0.01	0.04	0.79	-0.13	0.11	0.22	0.02	0.05	0.71	-0.03	0.06	0.56	0.04	0.08	0.60	-0.03	0.11	0.80
rs3817334	11	47650993	C	T	0.40	0.99	-	0.02	0.04	0.49	0.08	0.09	0.34	0.00	0.04	0.95	0.04	0.05	0.43	-0.04	0.06	0.53	-0.12	0.09	0.19
rs12286929	11	115022404	A	G	0.51	0.99	Genotyped	0.01	0.03	0.89	-0.02	0.09	0.83	0.00	0.04	0.95	0.05	0.05	0.26	0.04	0.06	0.50	0.09	0.09	0.32
rs7138803	12	50247468	G	A	0.38	1.00	Genotyped	0.03	0.04	0.38	0.03	0.09	0.73	0.03	0.04	0.40	0.01	0.05	0.89	0.12	0.07	0.06	-0.04	0.09	0.68
rs11057405	12	122781897	G	A	0.09	0.98	Genotyped	0.01	0.06	0.92	0.17	0.16	0.28	-0.03	0.07	0.68	0.05	0.08	0.53	0.02	0.11	0.82	0.42	0.18	0.02
rs9581854	13	28017782	C	T	0.17	0.84	-	-0.05	0.05	0.29	0.18	0.13	0.17	-0.10	0.06	0.08	-0.02	0.07	0.81	0.00	0.09	0.96	-0.20	0.12	0.10
rs12429545	13	54102206	G	A	0.13	1.00	Genotyped	0.03	0.05	0.60	0.09	0.13	0.47	-0.05	0.06	0.41	0.09	0.07	0.22	-0.02	0.09	0.87	0.07	0.14	0.62
rs9540493	13	66205704	A	G	0.51	0.37	-	-0.03	0.06	0.60	-0.10	0.14	0.48	-0.04	0.07	0.51	0.08	0.08	0.34	0.00	0.10	0.99	-0.03	0.15	0.82
rs1441264	13	79580919	G	A	0.62	0.20	-	0.01	0.08	0.89	-0.13	0.20	0.51	0.06	0.09	0.52	0.12	0.11	0.26	0.04	0.14	0.78	0.18	0.20	0.39
rs10132280	14	25928179	C	A	0.29	0.16	-	-0.10	0.09	0.30	0.38	0.25	0.13	-0.22	0.11	0.04	-0.14	0.13	0.30	0.23	0.18	0.20	0.16	0.25	0.51
rs12885454	14	29736838	C	A	0.30	0.09	-	0.18	0.13	0.16	0.39	0.33	0.24	0.14	0.15	0.35	0.22	0.18	0.23	0.20	0.24	0.40	0.36	0.35	0.31
rs11847697	14	30515112	C	T	0.05	0.99	Genotyped	-0.01	0.08	0.88	0.11	0.23	0.64	0.01	0.09	0.96	0.04	0.12	0.71	-0.15	0.16	0.34	-0.50	0.18	0.01
rs7141420	14	79899454	C	T	0.50	0.43	-	0.03	0.05	0.57	0.08	0.13	0.53	0.03	0.06	0.66	0.02	0.07	0.77	0.01	0.09	0.90	0.07	0.14	0.60
rs3736485	15	51748610	A	G	0.48	0.84	-	0.02	0.04	0.54	0.11	0.10	0.27	0.03	0.04	0.56	0.02	0.05	0.73	0.00	0.07	0.96	0.02	0.10	0.82
rs16951275	15	68077168	T	C	0.30	0.79	-	0.02	0.04	0.63	-0.10	0.10	0.35	0.02	0.05	0.68	0.00	0.06	0.95	0.07	0.08	0.40	0.10	0.11	0.38
rs7164727	15	73093991	C	T	0.64	0.94	-	-0.03	0.04	0.49	-0.03	0.10	0.75	-0.03	0.04	0.47	0.03	0.05	0.56	-0.09	0.07	0.21	0.09	0.10	0.34
rs758747	16	3627358	C	T	0.29	0.99	Genotyped	0.00	0.04	0.95	-0.16	0.09	0.10	0.04	0.04	0.40	-0.03	0.05	0.58	0.06	0.07	0.42	-0.11	0.10	0.25
rs12446632	16	19935389	G	A	0.12	0.98	-	0.00	0.05	0.98	-0.02	0.13	0.88	0.01	0.06	0.81	0.01	0.07	0.84	0.17	0.10	0.08	-0.01	0.13	0.91
rs2650492	16	28333411	G	A	0.27	0.95	-	0.04	0.04	0.28	0.16	0.10	0.10	0.01	0.04	0.88	0.09	0.05	0.08	0.05	0.07	0.47	-0.03	0.10	0.79
rs3888190	16	28889486	C	A	0.38	0.99	-	-0.02	0.04	0.61	0.20	0.09	0.02	-0.06	0.04	0.12	0.02	0.05	0.76	-0.07	0.06	0.26	-0.07	0.09	0.45
rs4787491	16	30015337	A	G	0.52	0.99	-	0.02	0.03	0.52	-0.04	0.09	0.69	0.03	0.04	0.45	-0.01	0.05	0.83	0.06	0.06	0.33	-0.10	0.09	0.30
rs9925964	16	31129895	A	G	0.42	0.95	-	0.04	0.04	0.28	0.05	0.09	0.60	0.02	0.04	0.60	0.03	0.05	0.60	0.07	0.07	0.28	0.04	0.10	0.69
rs2080454	16	49062590	C	A	0.63	0.55	-	-0.06	0.05	0.23	0.04	0.12	0.78	-0.08	0.06	0.13	-0.13	0.07	0.05	-0.10	0.09	0.24	-0.04	0.13	0.75
rs1558902	16	53803574	T	A	0.61	1.00	-	0.04	0.03	0.23	0.02	0.09	0.85	0.07	0.04	0.08	0.06	0.05	0.20	-0.03	0.06	0.59	0.16	0.09	0.09
rs9914578	17	2005136	C	G	0.22	0.69	-	-0.04	0.05	0.40	0.03	0.13	0.84	-0.06	0.06	0.32	0.03	0.07	0.64	-0.08	0.09	0.38	0.05	0.14	0.71
rs1000940	17	5283252	A	G	0.33	0.76	-	-0.03	0.04	0.40	-0.03	0.11	0.74	-0.02	0.05	0.70	-0.05	0.06	0.44	-0.04	0.08	0.65	0.13	0.12	0.28
rs12940622	17	78615571	G	A	0.42	0.46	-	0.09	0.05	0.08	0.10	0.13	0.45	0.11	0.06	0.06	0.00	0.07	0.98	0.20	0.09	0.03	0.24	0.13	0.07
rs1808579	18	21104888	C	T	0.48	0.97	-	0.00	0.04	0.93	0.04	0.09	0.66	0.01	0.04	0.80	0.01	0.05	0.82	0.05	0.06	0.42	-0.15	0.09	0.11
rs7239883	18	40147671	G	A	0.64	0.41	-	0.01	0.05	0.92	0.10	0.13	0.45	-0.04	0.06	0.50	-0.05	0.08	0.53	0.07	0.10	0.48	-0.07	0.14	0.62
rs7243357	18	56883319	T	G	0.18	0.97	-	0.04	0.04	0.32	0.03	0.11	0.80	0.02	0.05	0.72	0.06	0.06	0.32	0.10	0.08	0.21	-0.13	0.11	0.25
rs6567160	18	57829135	T	C	0.24	0.94	-	-0.04	0.04	0.29	0.10	0.11	0.34	-0.07	0.05	0.17	-0.06	0.06	0.30	-0.05	0.08	0.48	-0.20	0.10	0.05
rs17724992	19	18454825	A	G	0.32	0.46	-	0.08	0.05	0.14	0.26	0.14	0.07	0.10	0.06	0.10	0.24	0.08	0.00	-0.01	0.10	0.93	0.00	0.15	0.98
rs29941	19	34309532	A	G	0.63	0.65	-	-0.04	0.05	0.37	0.05	0.12	0.66	-0.09	0.05	0.07	-0.05	0.06	0.38	-0.10	0.08	0.23	0.02	0.12	0.89
rs2075650	19	45395619	A	G	0.15	0.44	-	0.02	0.07	0.76	0.27	0.19	0.15	-0.04	0.08	0.63	0.06	0.10	0.59	0.04	0.13	0.77	0.34	0.21	0.11
rs2287019	19	46202172	C	T	0.20	1.00	Genotyped	0.08	0.04	0.08	-0.09	0.11	0.39	0.10	0.05	0.04	0.03	0.06	0.58	0.13	0.08	0.10	0.25	0.12	0.05
rs3810291	19	47569003	G	A	0.64	0.92	-	0.02	0.04	0.53	-0.01	0.10	0.91	0.06	0.04	0.19	0.02	0.05	0.75	0.03	0.07	0.66	-0.08	0.10	0.46
rs6091540	20	51087862	C	T	0.29	0.97	-	-0.04	0.04	0.35	-0.08	0.09	0.42	-0.05	0.04	0.31	-0.10	0.05	0.07	-0.01	0.07	0.85	-0.03	0.10	0.76
rs2836754	21	40291740	T	C	0.59	0.99	Genotyped	0.01	0.03	0.69	-0.10	0.09	0.26	0.03	0.04	0.42	-0.06	0.05	0.20	-0.01	0.06	0.83	0.03	0.09	0.74

Supplementary Table S5 - Epic study rs number, chromosome and base position in hg19, genotyping and imputation information, and association parameters for analyzed BMI SNPs

								Overall									Adenocarcinoma			Squamous Cell			Small Cell		
								All sample			Never smokers			Ever smokers			All sample			All sample			All sample		
SNP	CHR	BP	EA	OA	Freq1	Info	Genotyped	Effect	SE	P	Effect	SE	P	Effect	SE	P	Effect	SE	P	Effect	SE	P	Effect	SE	P
rs977747	1	47684677	T	G	0.63	0.99	-	0.05	0.06	0.41	0.03	0.14	0.84	0.04	0.06	0.51	0.10	0.08	0.21	0.05	0.10	0.57	-0.02	0.11	0.88
rs657452	1	49589847	A	G	0.61	1.00	Genotyped	0.04	0.06	0.42	-0.06	0.14	0.63	0.07	0.06	0.31	0.03	0.08	0.75	0.09	0.09	0.35	-0.11	0.11	0.32
rs11583200	1	50559820	C	T	0.61	1.00	Genotyped	0.04	0.05	0.49	0.15	0.13	0.26	0.01	0.06	0.90	0.00	0.08	0.97	0.10	0.09	0.29	-0.11	0.11	0.29
rs3101336	1	72751185	T	C	0.62	0.99	-	0.07	0.06	0.24	0.07	0.14	0.65	0.06	0.06	0.35	0.11	0.08	0.17	-0.06	0.10	0.52	0.16	0.11	0.15
rs12566985	1	75002193	G	A	0.57	1.00	Genotyped	0.07	0.05	0.19	0.00	0.14	0.99	0.11	0.06	0.08	0.11	0.08	0.13	-0.12	0.09	0.19	0.20	0.11	0.06
rs12401738	1	78446761	G	A	0.32	1.00	Genotyped	-0.03	0.06	0.61	0.08	0.14	0.60	-0.05	0.07	0.48	-0.06	0.08	0.47	0.04	0.10	0.67	0.00	0.11	0.98
rs11165643	1	96924097	C	T	0.57	1.00	Genotyped	0.00	0.05	0.98	-0.15	0.14	0.28	0.09	0.06	0.17	0.00	0.08	0.98	0.08	0.10	0.38	0.05	0.11	0.64
rs17024393	1	110154688	T	C	0.03	1.00	Genotyped	0.02	0.16	0.90	0.81	0.56	0.15	-0.06	0.19	0.73	0.15	0.24	0.51	0.44	0.33	0.19	-0.60	0.26	0.02
rs543874	1	177889480	A	G	0.18	1.00	Genotyped	0.05	0.07	0.45	0.16	0.18	0.39	0.05	0.08	0.52	0.17	0.10	0.08	0.00	0.12	0.99	-0.06	0.13	0.62
rs2820292	1	201784287	A	C	0.55	0.99	-	-0.07	0.05	0.18	-0.04	0.13	0.75	-0.04	0.06	0.56	-0.05	0.07	0.52	-0.20	0.09	0.03	0.04	0.10	0.67
rs13021737	2	632348	A	G	0.82	0.98	-	0.02	0.07	0.74	-0.03	0.18	0.88	0.08	0.08	0.34	0.03	0.10	0.72	0.06	0.12	0.62	-0.01	0.14	0.97
rs10182181	2	25150296	A	G	0.45	1.00	Genotyped	0.08	0.05	0.13	0.07	0.13	0.60	0.07	0.06	0.26	0.08	0.07	0.29	0.08	0.09	0.38	0.06	0.10	0.55
rs11126666	2	26928811	G	A	0.27	1.00	Genotyped	-0.11	0.06	0.06	0.10	0.15	0.51	-0.17	0.07	0.01	-0.03	0.08	0.74	-0.14	0.10	0.16	-0.18	0.11	0.12
rs1016287	2	59305625	T	C	0.71	1.00	Genotyped	-0.04	0.06	0.49	-0.18	0.15	0.23	-0.03	0.07	0.62	-0.06	0.08	0.44	-0.05	0.10	0.60	-0.06	0.12	0.62
rs11688816	2	63053048	G	A	0.51	1.00	Genotyped	-0.02	0.05	0.66	-0.06	0.14	0.65	0.00	0.06	0.96	0.06	0.08	0.45	0.04	0.09	0.64	-0.18	0.11	0.09
rs2121279	2	143043285	C	T	0.12	1.00	Genotyped	-0.02	0.08	0.82	-0.14	0.19	0.47	0.01	0.09	0.93	-0.03	0.11	0.77	0.01	0.14	0.95	-0.01	0.16	0.93
rs1460676	2	164567689	T	C	0.15	0.80	-	-0.14	0.08	0.09	-0.24	0.21	0.26	-0.08	0.10	0.39	-0.05	0.12	0.66	-0.16	0.14	0.25	-0.05	0.16	0.75
rs1528435	2	181550962	C	T	0.88	0.29	-	-0.19	0.15	0.20	0.06	0.36	0.87	-0.27	0.17	0.12	0.05	0.20	0.82	-0.19	0.26	0.47	-0.28	0.30	0.34
rs17203016	2	208255518	A	G	0.18	1.00	Genotyped	-0.01	0.07	0.92	-0.05	0.17	0.75	-0.02	0.08	0.78	0.05	0.10	0.61	0.03	0.12	0.78	-0.03	0.14	0.80
rs7599312	2	213413231	G	A	0.25	1.00	Genotyped	0.02	0.06	0.69	-0.08	0.16	0.63	0.06	0.07	0.39	0.00	0.08	0.98	0.06	0.11	0.58	0.13	0.12	0.30
rs492400	2	219349752	C	T	0.58	0.99	-	-0.09	0.05	0.11	0.09	0.14	0.53	-0.11	0.06	0.06	-0.18	0.07	0.02	-0.01	0.09	0.95	-0.18	0.11	0.09
rs2176040	2	227092802	A	G	0.62	1.00	-	-0.02	0.05	0.79	0.02	0.14	0.90	-0.04	0.06	0.52	0.03	0.08	0.68	-0.09	0.10	0.33	-0.19	0.11	0.08
rs6804842	3	25106437	A	G	0.58	1.00	Genotyped	-0.02	0.05	0.76	0.16	0.14	0.23	-0.06	0.06	0.33	-0.04	0.08	0.63	-0.05	0.09	0.61	-0.04	0.11	0.70
rs2365389	3	61236462	C	T	0.40	0.98	-	-0.10	0.05	0.07	-0.19	0.14	0.16	-0.10	0.06	0.12	-0.23	0.08	0.00	-0.06	0.10	0.56	-0.03	0.11	0.77
rs3849570	3	81792112	C	A	0.32	1.00	-	-0.05	0.06	0.43	-0.31	0.14	0.03	0.00	0.07	0.99	-0.19	0.08	0.01	-0.03	0.10	0.79	0.08	0.11	0.47
rs13078960	3	85807590	T	G	0.19	1.00	Genotyped	0.20	0.07	0.00	0.27	0.17	0.11	0.13	0.08	0.11	0.19	0.10	0.05	0.01	0.12	0.93	0.25	0.14	0.07
rs16851483	3	141275436	G	T	0.06	0.97	-	-0.05	0.11	0.66	-0.35	0.27	0.19	0.03	0.13	0.84	-0.05	0.16	0.74	-0.15	0.19	0.43	-0.15	0.22	0.48
rs1516725	3	185824004	T	C	0.86	0.96	-	-0.03	0.08	0.70	0.19	0.19	0.33	-0.11	0.09	0.24	-0.11	0.11	0.34	0.03	0.13	0.84	-0.11	0.16	0.48
rs10938397	4	45182527	A	G	0.42	0.99	-	-0.05	0.05	0.40	0.07	0.14	0.62	-0.06	0.06	0.35	-0.08	0.08	0.31	0.03	0.10	0.75	-0.08	0.11	0.43
rs17001654	4	77129568	C	G	0.84	1.00	Genotyped	0.18	0.07	0.02	-0.02	0.19	0.92	0.25	0.09	0.00	0.20	0.10	0.05	0.26	0.12	0.03	0.33	0.13	0.01
rs13107325	4	103188709	C	T	0.07	1.00	Genotyped	0.04	0.10	0.68	-0.06	0.23	0.79	0.01	0.12	0.95	0.21	0.15	0.18	0.12	0.19	0.51	-0.06	0.19	0.75
rs11727676	4	145659064	T	C	0.09	1.00	Genotyped	0.02	0.09	0.81	-0.23	0.24	0.34	0.09	0.11	0.39	-0.03	0.13	0.83	0.22	0.17	0.20	-0.13	0.18	0.45
rs2112347	5	75015242	T	G	0.37	0.96	-	0.01	0.06	0.92	0.01	0.14	0.94	0.01	0.06	0.92	0.11	0.08	0.17	0.01	0.10	0.89	-0.15	0.11	0.17
rs7715256	5	153537893	G	T	0.59	0.96	-	0.02	0.05	0.78	0.08	0.14	0.55	0.01	0.06	0.91	0.11	0.08	0.14	-0.11	0.09	0.26	-0.04	0.11	0.68
rs205262	6	34563164	A	G	0.29	1.00	Genotyped	0.06	0.06	0.33	0.37	0.15	0.02	0.00	0.07	0.99	0.02	0.08	0.84	0.03	0.10	0.79	0.10	0.12	0.40
rs2033529	6	40348653	A	G	0.28	1.00	Genotyped	-0.02	0.06	0.74	-0.19	0.15	0.19	0.00	0.07	1.00	-0.04	0.08	0.61	-0.05	0.10	0.63	-0.13	0.11	0.25
rs2207139	6	50845490	A	G	0.17	1.00	-	0.00	0.07	0.97	-0.17	0.17	0.32	0.03	0.08	0.73	0.03	0.10	0.78	-0.20	0.12	0.09	-0.07	0.14	0.62
rs9400239	6	108977663	T	C	0.69	0.98	-	0.03	0.06	0.66	0.19	0.15	0.20	0.01	0.07	0.88	0.04	0.08	0.60	-0.13	0.11	0.23	-0.11	0.12	0.36
rs9374842	6	120185665	C	T	0.77	0.98	-	-0.01	0.06	0.93	0.03	0.16	0.85	0.02	0.07	0.79	-0.04	0.09	0.68	-0.01	0.11	0.95	-0.12	0.13	0.37
rs13201877	6	137675541	A	G	0.13	1.00	Genotyped	0.03	0.08	0.74	-0.11	0.20	0.57	0.08	0.09	0.37	-0.03	0.11	0.76	0.08	0.14	0.59	0.01	0.16	0.94
rs13191362	6	163033350	A	G	0.14	1.00	Genotyped	0.06	0.08	0.47	-0.25	0.18	0.17	0.09	0.09	0.35	-0.03	0.11	0.76	0.25	0.14	0.08	-0.01	0.15	0.97
rs1167827	7	75163169	A	G	0.57	1.00	Genotyped	0.00	0.05	0.98	0.14	0.13	0.30	-0.01	0.06	0.87	0.00	0.07	0.99	0.13	0.09	0.16	-0.12	0.11	0.27
rs2245368	7	76608143	C	T	0.82	0.99	Genotyped	-0.06	0.07	0.39	-0.16	0.18	0.39	-0.06	0.08	0.42	0.00	0.09	0.99	-0.06	0.12	0.62	0.17	0.13	0.19
rs9641123	7	93197732	G	C	0.41	1.00	Genotyped	-0.02	0.05	0.72	0.03	0.13	0.82	-0.01	0.06	0.88	-0.03	0.08	0.72	0.03	0.09	0.78	-0.06	0.11	0.57
rs6465468	7	95169514	G	T	0.30	1.00	Genotyped	-0.02	0.06	0.70	0.12	0.15	0.41	-0.03	0.07	0.69	0.00	0.08	0.98	-0.09	0.10	0.39	-0.12	0.11	0.30
rs17405819	8	76806584	T	C	0.31	1.00	-	0.04	0.06	0.52	0.03	0.14	0.81	0.06	0.07	0.34	-0.01	0.08	0.87	0.07	0.10	0.48	0.20	0.12	0.08
rs16907751	8	81375457	C	T	0.11	1.00	Genotyped	-0.05	0.09	0.53	0.05	0.21	0.80	-0.07	0.10	0.46	0.06	0.12	0.61	-0.23	0.14	0.11	-0.16	0.16	0.33
rs2033732	8	85079709	T	C	0.76	1.00	Genotyped	-0.07	0.06	0.25	-0.11	0.16	0.48	-0.04	0.07	0.60	-0.07	0.09	0.43	-0.06	0.11	0.56	-0.18	0.13	0.14
rs4740619	9	15634326	T	C	0.46	1.00	-	-0.03	0.05	0.57	-0.17	0.14	0.21	-0.03	0.06	0.67	0.01	0.07	0.88	-0.08	0.09	0.38	0.03	0.10	0.81
rs10968576	9	28414339	A	G	0.29	1.00	-	0.03	0.06	0.66	-0.09	0.15	0.55	0.04	0.07	0.54	0.10	0.08	0.22	-0.05	0.10	0.65	-0.05	0.11	0.68
rs6477694	9	111932342	C	T	0.65	1.00	Genotyped	0.05	0.06	0.41	-0.05	0.14	0.73	0.06	0.06	0.34	0.04	0.08	0.61	0.11	0.09	0.24	-0.02	0.11	0.89
rs1928295	9	120378483	T	C	0.45	1.00	Genotyped	0.08	0.05	0.12	0.11	0.13	0.40	0.09	0.06	0.14	0.13	0.08	0.08	0.09	0.09	0.36	0.14	0.11	0.20
rs10733682	9	129460914	A	G	0.52	1.00	Genotyped	0.00	0.05	0.95	0.04	0.13	0.78	-0.05	0.06	0.41	-0.07	0.07	0.34	0.10	0.09	0.27	0.14	0.10	0.17
rs7899106	10	87410904	A	G	0.04	1.00	Genotyped	0.09	0.13	0.47	0.22	0.35	0.52	0.11	0.15	0.46	0.04	0.18	0.82	-0.01	0.22	0.96	0.15	0.26	0.57
rs17094222	10	102395440	T	C	0.21	1.00	Genotyped	0.07	0.07	0.30	0.16	0.16	0.33	0.01	0.08	0.88	0.00	0.09	0.99	0.12	0.12	0.32	0.16	0.13	0.22
rs11191560	10	104869038	T	C	0.09	0.99	-	-0.12	0.09	0.19	-0.20	0.23	0.39	-0.06	0.11	0.55	-0.19	0.12	0.13	-0.07	0.16	0.68	-0.21	0.17	0.23
rs7903146	10	114758349	C	T	0.31	1.00	Genotyped	-0.01	0.06	0.86	0.02	0.15	0.88	-0.01	0.07	0.87	-0.02	0.08	0.82	-0.07	0.10	0.53	0.13	0.12	0.27
rs4256980	11	8673939	C	G	0.37	1.00	Genotyped	0.03	0.06	0.63	0.05	0.14	0.71	0.05	0.06	0.49	0.11	0.08	0.15	-0.04	0.09	0.66	-0.15	0.10	0.16
rs11030104	11	27684517	A	G	0.22	0.99	-	0.02	0.06	0.77	0.07	0.16	0.68	0.02	0.08	0.79	0.02	0.09	0.81	0.11	0.11	0.34	0.14	0.13	0.29
rs2176598	11	43864278	T	C	0.75	1.00	Genotyped	-0.05	0.06	0.43	-0.55	0.17	0.00	0.05	0.07	0.45	-0.19	0.09	0.03	-0.06	0.11	0.60	0.04	0.12	0.72
rs3817334	11	47650993	C	T	0.40	0.99	-	0.08	0.05	0.14	0.42	0.14	0.00	0.03	0.06	0.68	0.11	0.08	0.15	0.02	0.09	0.81	-0.04	0.10	0.68
rs12286929	11	115022404	A	G	0.54	1.00	Genotyped	-0.03	0.05	0.61	-0.03	0.14	0.86	-0.06	0.06	0.31	0.02	0.08	0.85	-0.10	0.09	0.31	0.11	0.11	0.29
rs7138803	12	50247468	G	A	0.38	1.00	Genotyped	0.02	0.06	0.70	-0.16	0.13	0.25	0.05	0.06	0.43	0.01	0.08	0.91	0.05	0.10	0.58	0.21	0.11	0.06
rs11057405	12	122781897	G	A	0.09	1.00	Genotyped	0.05	0.09	0.60	0.15	0.25	0.54	0.05	0.10	0.64	0.25	0.14	0.07	-0.27	0.14	0.06	0.32	0.20	0.11
rs9581854	13	28017782	C	T	0.20	0.99	-	0.01	0.07	0.89	-0.01	0.16	0.95	-0.04	0.08	0.57	0.03	0.09	0.74	-0.02	0.11	0.83	-0.13	0.13	0.31
rs12429545	13	54102206	G	A	0.12	1.00	Genotyped	0.01	0.08	0.87	0.18	0.21	0.39	-0.06	0.09	0.53	0.11	0.12	0.35	0.18	0.15	0.22	-0.22	0.15	0.13
rs9540493	13	66205704	A	G	0.57	1.00	Genotyped	0.02	0.05	0.75	0.04	0.14	0.79	-0.03	0.06	0.66	-0.02	0.08	0.78	-0.11	0.09	0.25	0.02	0.11	0.84
rs1441264	13	79580919	G	A	0.62	1.00	Genotyped	0.09	0.06	0.11	0.20	0.14	0.13	0.07	0.06	0.29	0.15	0.08	0.05	0.05	0.10	0.63	0.09	0.11	0.42
rs10132280	14	25928179	C	A	0.30	0.94	-	0.08	0.06	0.21	0.10	0.15	0.52	0.03	0.07	0.62	0.04	0.08	0.65	0.26	0.11	0.02	0.04	0.12	0.71
rs12885454	14	29736838	C	A	0.35	0.99	-	-0.04	0.06	0.46	0.09	0.14	0.53	-0.04	0.06	0.53	-0.10	0.08	0.17	-0.06	0.10	0.53	0.03	0.11	0.82
rs11847697	14	30515112	C	T	0.05	1.00	Genotyped	-0.03	0.13	0.79	0.33	0.35	0.34	-0.06	0.15	0.66	0.09	0.19	0.64	-0.16	0.21	0.44	-0.26	0.23	0.26
rs7141420	14	79899454	C	T	0.52	1.00	Genotyped	-0.07	0.05	0.18	0.11	0.13	0.42	-0.11	0.06	0.09	0.00	0.07	0.99	-0.12	0.09	0.21	-0.06	0.10	0.56
rs3736485	15	51748610	A	G	0.46	0.87	-	-0.02	0.06	0.73	0.21	0.15	0.16	-0.07	0.07	0.27	0.03	0.08	0.71	-0.15	0.10	0.14	-0.18	0.11	0.10
rs16951275	15	68077168	T	C	0.22	1.00	-	0.00	0.06	1.00	0.21	0.16	0.19	-0.05	0.07	0.48	0.04	0.09	0.69	0.04	0.11	0.73	0.10	0.12	0.42
rs7164727	15	73093991	C	T	0.68	1.00	Genotyped	-0.08	0.06	0.15	0.04	0.14	0.81	-0.09	0.07	0.17	-0.02	0.08	0.82	-0.11	0.10	0.29	-0.11	0.11	0.31
rs758747	16	3627358	C	T	0.28	0.65	-	0.01	0.07	0.94	-0.18	0.19	0.34	0.06	0.09	0.51	-0.07	0.10	0.52	-0.01	0.13	0.95	0.05	0.15	0.71
rs12446632	16	19935389	G	A	0.13	1.00	Genotyped	0.15	0.08	0.05	-0.13	0.19	0.50	0.21	0.09	0.02	0.12	0.11	0.29	0.30	0.14	0.04	0.09	0.15	0.56
rs2650492	16	28333411	G	A	0.29	1.00	Genotyped	0.01	0.06	0.93	0.29	0.16	0.07	-0.02	0.07	0.78	0.05	0.08	0.51	-0.08	0.10	0.40	0.02	0.11	0.89
rs3888190	16	28889486	C	A	0.38	0.98	-	-0.01	0.06	0.88	0.11	0.15	0.46	-0.02	0.06	0.76	0.07	0.08	0.40	-0.18	0.10	0.06	0.00	0.11	0.99
rs4787491	16	30015337	A	G	0.52	1.00	Genotyped	0.14	0.05	0.01	0.15	0.14	0.29	0.13	0.06	0.03	0.06	0.07	0.43	0.33	0.09	0.00	0.11	0.10	0.31
rs9925964	16	31129895	A	G	0.38	1.00	Genotyped	0.01	0.06	0.87	0.08	0.14	0.57	-0.03	0.06	0.68	0.03	0.08	0.75	0.09	0.10	0.36	0.00	0.11	0.98
rs2080454	16	49062590	C	A	0.63	1.00	Genotyped	-0.08	0.06	0.17	0.09	0.14	0.52	-0.11	0.06	0.07	-0.02	0.08	0.79	0.01	0.09	0.94	-0.12	0.11	0.27
rs1558902	16	53803574	T	A	0.58	1.00	Genotyped	-0.02	0.05	0.71	0.02	0.13	0.85	0.00	0.06	0.99	0.04	0.08	0.61	0.06	0.09	0.53	-0.08	0.11	0.46
rs9914578	17	2005136	C	G	0.21	0.99	-	-0.11	0.07	0.11	0.06	0.17	0.73	-0.14	0.08	0.07	-0.26	0.09	0.00	-0.08	0.11	0.50	-0.07	0.13	0.58
rs1000940	17	5283252	A	G	0.31	0.99	-	-0.02	0.06	0.73	0.10	0.14	0.50	-0.06	0.07	0.39	0.03	0.08	0.69	0.01	0.10	0.89	-0.08	0.11	0.51
rs12940622	17	78615571	G	A	0.43	1.00	Genotyped	-0.01	0.05	0.80	-0.25	0.13	0.06	0.06	0.06	0.35	-0.01	0.08	0.92	0.09	0.09	0.36	-0.01	0.11	0.93
rs1808579	18	21104888	C	T	0.43	1.00	Genotyped	0.02	0.05	0.76	0.11	0.14	0.42	0.01	0.06	0.93	-0.05	0.08	0.55	0.00	0.09	0.98	0.12	0.11	0.24
rs7239883	18	40147671	G	A	0.60	1.00	Genotyped	0.07	0.05	0.23	-0.06	0.13	0.65	0.08	0.06	0.19	0.07	0.08	0.34	0.13	0.09	0.16	0.14	0.11	0.19
rs7243357	18	56883319	T	G	0.18	1.00	Genotyped	-0.10	0.07	0.18	-0.36	0.17	0.03	-0.03	0.08	0.74	-0.06	0.10	0.51	-0.06	0.12	0.65	-0.08	0.13	0.58
rs6567160	18	57829135	T	C	0.25	1.00	-	0.04	0.06	0.55	0.45	0.17	0.01	0.00	0.07	0.99	0.29	0.09	0.00	-0.05	0.11	0.61	-0.16	0.12	0.19
rs17724992	19	18454825	A	G	0.26	1.00	Genotyped	0.15	0.06	0.01	0.27	0.16	0.10	0.13	0.07	0.08	0.20	0.09	0.02	0.13	0.11	0.23	0.15	0.12	0.23
rs29941	19	34309532	G	A	0.69	0.98	-	0.04	0.06	0.51	0.11	0.14	0.44	0.01	0.07	0.85	0.02	0.08	0.80	0.05	0.10	0.66	-0.11	0.12	0.33
rs2075650	19	45395619	A	G	0.13	1.00	Genotyped	-0.08	0.08	0.34	0.40	0.22	0.08	-0.18	0.09	0.05	-0.04	0.11	0.75	-0.13	0.13	0.35	-0.02	0.16	0.90
rs2287019	19	46202172	C	T	0.20	1.00	Genotyped	-0.07	0.07	0.30	-0.13	0.16	0.40	-0.06	0.08	0.42	-0.04	0.09	0.70	-0.01	0.12	0.96	-0.03	0.13	0.84
rs3810291	19	47569003	G	A	0.68	1.00	Genotyped	0.00	0.06	0.98	-0.03	0.14	0.81	-0.01	0.07	0.83	-0.03	0.08	0.72	-0.02	0.10	0.85	0.09	0.11	0.43
rs6091540	20	51087862	C	T	0.27	1.00	-	0.02	0.06	0.74	-0.01	0.15	0.95	0.03	0.07	0.66	0.05	0.08	0.58	0.07	0.11	0.49	-0.14	0.11	0.22
rs2836754	21	40291740	T	C	0.64	1.00	Genotyped	-0.07	0.06	0.24	0.01	0.14	0.94	-0.05	0.06	0.43	-0.09	0.08	0.28	-0.05	0.10	0.57	-0.13	0.11	0.22

Supplementary Table S6 - Meta-analyses and study regression analyses of genetic BMI score over phenotypic BMI, pack years (PY), cigarettes per day (CPD) and cotinine levels in the EPIC-OncoArray sample; in the whole sample and within cases and controls. Regression models were adjusted by gender and age and principal components for population stratification. I2: heterogeneity index. R2: proporion of phenotypic variance explained by the variables included in the regression model.

Study	Phenotype	Sample	N	Estimate	LIC	UIC	P	I^2
**Pooled results**	**BMI**	Overall	16,703	3.27	2.70	3.84	<1.0E-17	0.44
		Controls	9,093	3.22	2.44	4.01	8.88E-16	0.13
		Cases	7,430	3.28	2.45	4.12	1.40E-14	0
	**Pack years**	Overall	6,518	2.27	-1.13	5.68	0.19	0.18
		Controls	2,827	0.79	-4.26	5.83	0.76	0
		Cases	3,609	3.18	-1.39	7.76	0.17	0.21
	**CPD**	Overall	1,268	1.65	-1.93	5.23	0.37	
		Controls	416	3.29	-2.98	9.55	0.30	
		Cases	852	0.59	-3.71	4.89	0.79	
	**Cotinine**	Overall	505	94.20	-227.89	416.29	0.57	
		Controls	143	34.00	-528.50	596.51	0.91	
		Cases	362	28.32	-340.57	397.20	0.88	
**IARC-ILCCO**	**BMI**	Overall	6,327	3.53	2.36	4.70	3.67E-09	
		Controls	3,790	3.96	2.37	5.55	1.19E-06	
		Cases	2,537	3.18	1.47	4.89	2.68E-04	
	**Pack years**	Overall	3,470	3.27	-0.56	7.11	0.09	
		Controls	1,579	1.36	-4.39	7.12	0.64	
		Cases	1,891	4.47	-0.63	9.57	0.09	
**Axiom**	**BMI**	Overall	7,486	3.84	2.88	4.80	4.49E-15	
		Controls	3,850	3.51	2.21	4.81	1.22E-07	
		Cases	3,456	4.11	2.64	5.58	4.63E-08	
	**Pack years**	Overall	3,048	-1.42	-8.81	5.96	0.71	
		Controls	1,248	-1.12	-11.58	9.35	0.83	
		Cases	1,718	-2.17	-12.57	8.22	0.68	
**EPIC**	**BMI**	Overall	2,890	2.62	1.73	3.51	8.94E-09	
		Controls	1,453	2.49	1.24	3.75	1.01E-04	
		Cases	1,437	2.72	1.46	3.99	2.58E-05	
	**CPD**	Overall	1,268	1.65	-1.93	5.23	0.37	
		Controls	416	3.29	-2.98	9.55	0.30	
		Cases	852	0.59	-3.71	4.89	0.79	
	**Cotinine**	Overall	505	94.20	-227.89	416.29	0.57	
		Controls	143	34.00	-528.50	596.51	0.91	
		Cases	362	28.32	-340.57	397.20	0.88	

Supplementary Table S7 - Genetic effects on BMI provided by GIANT and association parameters of meta-analyses on overall lung, AD, SQ and SC subtypes for the 59 BMI SNPs. CHR: Chromosome. BP: Base pair postion. EA: Effect allele. OA: Other allele. P: P value. The order of the direction effects were TRICL or IARC-ILCCO, EPIC and Axiom. 0: null effect. *: non-available effect.

										Overall												Adenocarcinoma				Squamous Cell Carcinoma				Small Cell Carcinoma			
			GIANT data on BMI phenotype					Lung data		All sample				Never smokers				Ever smokers				All sample				All sample				All sample			
SNP	CHR	BP	EA	OA	Gene	Effect (β_{GP})	β_{GP}se	EA	OA	Effect (β_{GD})	β_{GD}se	P	Direction	Effect (β_{GD}	β_{GD}se	P	Direction	Effect (β_{GD}	β_{GD}se	P	Direction	Effect (β_{GD})	β_{GD}se	P	Direction	Effect (β_{GD})	β_{GD}se	P	Direction	Effect (β_{GD})	β_{GD}se	P	Direction
rs977747	1	47684677	T	G	TAL1	0.017	0.003	T	G	-0.033	0.02	0.05	-+*	-0.116	0.10	0.23	-+*	-0.012	0.04	0.74	-+*	-0.020	0.03	0.45	-+*	-0.039	0.03	0.13	-+*	-0.029	0.06	0.66	--*
rs657452	1	49589847	A	G	AGBL4	0.023	0.003	A	G	0.001	0.02	0.97	-+-	-0.001	0.07	0.99	--+	-0.009	0.03	0.74	-+-	0.009	0.02	0.72	++-	0.034	0.03	0.18	+++	-0.086	0.05	0.11	---
rs1158320(	1	50559820	C	T	*ELAVL4*	0.018	0.003	T	C	-0.004	0.02	0.82	--*	-0.131	0.09	0.15	--*	0.004	0.04	0.90	+-*	-0.010	0.03	0.70	--*	0.004	0.03	0.87	+-*	0.027	0.06	0.67	-+*
rs3101336	1	72751185	C	T	NEGR1	0.033	0.003	T	C	0.029	0.02	0.07	+++	-0.006	0.07	0.93	-++	0.037	0.03	0.17	+++	0.054	0.02	0.03	+++	0.038	0.03	0.14	+-+	0.072	0.05	0.18	++-
rs1256698!	1	75002193	G	A	*FPGT-TNNI3K*	0.024	0.003	A	G	0.014	0.03	0.65	*-+	0.050	0.07	0.46	--+	0.017	0.03	0.55	+-+	-0.010	0.04	0.81	*-+	0.104	0.05	0.05	*++	-0.114	0.06	0.04	---
rs1240173(	1	78446761	A	G	*FUBP1*	0.021	0.003	A	G	0.069	0.02	0.00	+++	0.027	0.08	0.72	+-+	0.112	0.03	0.00	+++	0.033	0.03	0.20	+++	0.091	0.03	0.00	+-+	0.158	0.06	0.01	+++
rs1116564:	1	96924097	T	C	*PTBP2*	0.022	0.003	T	C	-0.011	0.03	0.70	*+-	0.022	0.06	0.73	-+-	-0.025	0.03	0.35	+--	0.034	0.04	0.41	*-+	0.012	0.05	0.82	*-+	0.028	0.05	0.59	+-+
rs1702439:	1	110154688	C	T	*GNAT2*	0.066	0.009	T	C	0.035	0.04	0.38	+++	0.178	0.18	0.32	-++	0.058	0.07	0.39	+-+	0.027	0.06	0.67	-++	0.023	0.06	0.71	+++	-0.092	0.13	0.49	+-+
rs543874	1	177889480	G	A	SEC16B	0.048	0.004	A	G	0.006	0.02	0.76	++-	-0.067	0.08	0.40	-+-	-0.054	0.03	0.11	-+-	-0.011	0.03	0.69	++-	0.020	0.03	0.50	+--	-0.064	0.07	0.33	---
rs2820292	1	201784287	C	A	NAV1	0.020	0.003	A	C	-0.008	0.02	0.61	--+	0.054	0.07	0.42	--+	0.023	0.03	0.40	+-+	-0.010	0.02	0.66	--+	-0.006	0.02	0.83	--+	0.011	0.05	0.84	-++
rs1302173	2	632348	G	A	*TMEM18*	0.060	0.004	A	G	-0.010	0.02	0.60	-++	-0.063	0.08	0.44	---	0.041	0.03	0.24	+++	0.038	0.03	0.20	+++	-0.020	0.03	0.51	-++	-0.022	0.07	0.75	+--
rs1018218:	2	25150296	G	A	*ADCY3*	0.031	0.003	A	G	-0.019	0.02	0.21	-+-	0.010	0.06	0.87	-+-	-0.016	0.03	0.53	-+-	-0.010	0.02	0.66	-++	-0.031	0.03	0.22	-++	-0.004	0.05	0.94	++-
rs1112666(	2	26928811	A	G	*KCNK3*	0.021	0.003	A	G	0.009	0.02	0.61	-++	-0.023	0.07	0.75	+--	0.056	0.03	0.06	+++	-0.036	0.02	0.14	-++	0.013	0.03	0.63	++-	0.032	0.06	0.58	-++
rs1016287	2	59305625	T	C	*LINC01122*	0.023	0.003	T	C	-0.034	0.02	0.04	---	-0.103	0.09	0.24	*--	-0.048	0.04	0.21	*--	-0.063	0.02	0.01	---	-0.032	0.03	0.22	---	-0.070	0.08	0.38	*--
rs1168881(	2	63053048	G	A	*EHBP1*	0.017	0.003	A	G	0.017	0.02	0.28	+++	0.131	0.07	0.05	+++	0.064	0.03	0.02	+++	0.024	0.02	0.29	+-+	0.012	0.02	0.62	+-+	0.049	0.05	0.36	++-
rs2121279	2	143043285	T	C	*LRP1B*	0.025	0.004	T	C	0.022	0.03	0.38	++*	0.084	0.13	0.52	++*	0.044	0.05	0.37	+-*	-0.073	0.04	0.04	-+*	0.045	0.04	0.25	+-*	0.124	0.09	0.16	++*
rs1460676	2	164567689	C	T	*FIGN*	0.020	0.004	T	C	-0.025	0.02	0.27	--*	-0.080	0.13	0.52	+-*	-0.072	0.05	0.13	--*	-0.042	0.04	0.23	--*	-0.022	0.03	0.53	--*	-0.126	0.08	0.14	--*
rs1528435	2	181550962	T	C	*UBE2E3*	0.018	0.003	T	C	0.005	0.02	0.79	+**	0.133	0.13	0.30	+**	-0.065	0.04	0.14	-**	-0.006	0.03	0.84	-**	-0.004	0.03	0.88	-**	0.030	0.08	0.71	+**
rs1720301(	2	208255518	G	A	*CREB1*	0.021	0.004	A	G	-0.013	0.02	0.57	--*	0.017	0.12	0.89	+-*	0.016	0.05	0.73	+-*	-0.012	0.03	0.73	-+*	0.003	0.03	0.93	++*	-0.001	0.09	0.99	+-*
rs7599312	2	213413231	G	A	ERBB4	0.022	0.003	A	G	-0.001	0.02	0.93	--+	0.131	0.07	0.06	-++	-0.045	0.03	0.12	---	-0.010	0.03	0.69	--+	-0.027	0.03	0.30	---	-0.110	0.06	0.06	---
rs492400	2	219349752	C	T	USP37	0.016	0.003	T	C	0.040	0.02	0.01	++-	-0.116	0.07	0.08	---	0.048	0.03	0.07	+++	0.018	0.02	0.43	++-	0.064	0.02	0.01	+++	0.095	0.05	0.08	+++
rs2176040	2	227092802	A	G	*LOC646736*	0.014	0.003	A	G	-0.014	0.02	0.36	---	0.080	0.07	0.22	+++	-0.034	0.03	0.20	---	-0.001	0.02	0.96	-++	-0.030	0.02	0.23	--+	-0.082	0.05	0.13	---
rs6804842	3	25106437	G	A	RARB	0.019	0.003	A	G	0.021	0.02	0.24	+-*	0.072	0.10	0.46	-+*	-0.027	0.04	0.46	--*	0.029	0.03	0.29	+-*	0.011	0.03	0.69	+-*	-0.059	0.07	0.38	--*
rs2365389	3	61236462	C	T	*FHIT*	0.020	0.003	T	C	0.032	0.02	0.07	++*	0.059	0.09	0.53	-+*	0.065	0.04	0.07	++*	0.035	0.03	0.18	++*	0.013	0.03	0.63	++*	-0.001	0.07	0.99	-+*
rs3849570	3	81792112	A	C	GBE1	0.019	0.003	A	C	-0.007	0.02	0.69	++-	-0.001	0.07	0.99	++-	-0.007	0.03	0.82	+--	-0.020	0.02	0.41	-+-	0.018	0.03	0.49	++-	0.045	0.06	0.41	+--
rs1307896(	3	85807590	G	T	*CADM2*	0.030	0.004	T	G	0.010	0.02	0.61	-+-	0.078	0.08	0.33	++-	-0.003	0.03	0.94	-+-	0.002	0.03	0.95	-++	0.018	0.03	0.56	++-	-0.017	0.07	0.79	-+-
rs1685148:	3	141275436	T	G	*RASA2*	0.048	0.008	T	G	-0.016	0.03	0.60	++-	-0.059	0.12	0.63	-+-	-0.082	0.05	0.12	---	-0.048	0.04	0.29	-+-	0.019	0.05	0.70	++-	-0.120	0.11	0.26	-+-
rs1516725	3	185824004	C	T	*ETV5*	0.045	0.005	T	C	0.000	0.02	0.99	+--	0.039	0.10	0.70	++-	-0.048	0.04	0.24	+--	-0.013	0.03	0.70	+--	0.011	0.04	0.76	++-	-0.062	0.08	0.45	+--
rs1093839	4	45182527	G	A	*GNPDA2*	0.040	0.003	A	G	0.011	0.02	0.48	+-+	0.023	0.07	0.73	-++	-0.028	0.03	0.30	--+	0.046	0.02	0.04	+-+	0.017	0.02	0.49	+++	-0.091	0.05	0.09	--+
rs1700165	4	77129568	G	C	*SCARB2*	0.031	0.005	C	G	-0.015	0.02	0.50	-+-	0.067	0.09	0.46	+-+	0.015	0.04	0.69	-+-	-0.026	0.03	0.44	-+-	-0.012	0.04	0.75	-++	0.080	0.07	0.27	++-
rs1310732!	4	103188709	T	C	*SLC39A8*	0.048	0.007	T	C	0.047	0.03	0.11	+-+	0.057	0.12	0.63	+++	0.038	0.05	0.44	+-+	0.025	0.04	0.56	+-+	0.047	0.05	0.31	+-+	0.044	0.10	0.64	+++
rs1172767(	4	145659064	T	C	*HHIP*	0.036	0.006	T	C	0.030	0.03	0.33	+++	0.063	0.15	0.67	*-+	0.036	0.07	0.58	*++	0.008	0.05	0.86	+--	0.064	0.05	0.17	++-	0.052	0.14	0.70	*-+
rs2112347	5	75015242	T	G	*POC5*	0.026	0.003	T	G	0.006	0.02	0.69	++-	-0.158	0.07	0.02	-+-	0.023	0.03	0.45	-++	-0.011	0.02	0.66	-+-	-0.010	0.03	0.70	++-	-0.054	0.06	0.37	--+
rs7715256	5	153537893	G	T	GALNT10	0.016	0.003	T	G	0.023	0.02	0.18	+-*	-0.048	0.09	0.60	--*	0.004	0.04	0.91	+-*	0.033	0.02	0.18	+-*	0.022	0.03	0.41	++*	-0.044	0.06	0.49	-+*
rs205262	6	34563164	G	A	*C6orf106*	0.022	0.004	A	G	-0.005	0.02	0.79	-+*	0.191	0.10	0.06	++*	-0.004	0.04	0.92	-+*	0.004	0.03	0.88	++*	-0.019	0.03	0.52	-+*	0.056	0.07	0.43	++*
rs2033529	6	40348653	G	A	*TDRG1*	0.019	0.003	A	G	0.022	0.02	0.22	+-*	-0.180	0.10	0.07	--*	0.033	0.04	0.38	+0*	0.016	0.03	0.55	+-*	0.025	0.03	0.37	+-*	0.032	0.07	0.64	+-*
rs2207139	6	50845490	G	A	*TFAP2B*	0.045	0.004	A	G	-0.022	0.02	0.27	-+-	-0.119	0.08	0.14	---	-0.012	0.03	0.72	++-	0.017	0.03	0.56	++-	-0.067	0.03	0.04	---	-0.060	0.07	0.36	+--
rs9400239	6	108977663	C	T	FOXO3	0.019	0.003	T	C	0.011	0.02	0.51	+++	0.074	0.07	0.28	+++	0.044	0.03	0.12	+++	-0.008	0.02	0.75	-++	0.026	0.03	0.34	+--	0.037	0.06	0.52	--+
rs9374842	6	120185665	T	C	LOC285762	0.019	0.004	T	C	0.013	0.02	0.52	++*	-0.054	0.10	0.60	--*	-0.009	0.04	0.81	--*	0.011	0.03	0.72	++*	0.045	0.03	0.12	++*	-0.049	0.07	0.50	-+*
rs1320187	6	137675541	G	A	*IFNGR1*	0.023	0.005	A	G	0.012	0.02	0.63	++*	-0.042	0.13	0.76	+-*	0.036	0.05	0.49	++*	0.012	0.04	0.74	+-*	-0.024	0.04	0.54	-+*	0.087	0.10	0.37	++*
rs1319136:	6	163033350	A	G	*PARK2*	0.028	0.005	A	G	0.038	0.03	0.14	++*	-0.257	0.13	0.06	--*	0.082	0.06	0.14	++*	0.021	0.04	0.59	+-*	0.024	0.04	0.56	++*	0.008	0.10	0.94	+-*
rs1167827	7	75163169	G	A	*HIP1*	0.020	0.003	A	G	0.009	0.02	0.59	++*	0.041	0.09	0.65	-+*	0.037	0.03	0.29	+-*	0.019	0.03	0.47	++*	0.015	0.03	0.57	++*	0.054	0.06	0.39	+-*
rs2245368	7	76608143	C	T	*PMS2L11*	0.032	0.006	T	C	0.059	0.07	0.39	*+*	0.157	0.18	0.39	*+*	0.063	0.08	0.42	*+*	-0.002	0.09	0.98	*-*	0.060	0.12	0.62	*+*	-0.170	0.13	0.18	*-*
rs9641123	7	93197732	C	G	CALCR	0.019	0.004	C	G	0.020	0.05	0.71	*+*	-0.045	0.09	0.62	--*	0.028	0.04	0.43	++*	0.027	0.08	0.72	*+*	-0.026	0.09	0.78	*-*	-0.024	0.06	0.71	-+*
rs6465468	7	95169514	T	G	ASB4	0.017	0.004	T	G	0.014	0.02	0.46	++*	-0.003	0.10	0.97	+-*	-0.019	0.04	0.64	-+*	0.000	0.03	0.99	++*	0.031	0.03	0.29	++*	0.061	0.07	0.39	++*
rs1740581!	8	76806584	T	C	*HNF4G*	0.022	0.003	T	C	-0.008	0.02	0.62	-+-	-0.080	0.07	0.28	-+-	-0.023	0.03	0.45	-+-	0.013	0.02	0.61	+--	-0.008	0.03	0.76	-++	0.033	0.06	0.58	-++
rs1690775:	8	81375457	C	T	*ZBTB10*	0.035	0.007	T	C	0.036	0.03	0.15	++-	0.004	0.10	0.97	--+	-0.016	0.04	0.71	++-	0.037	0.04	0.31	+-+	0.088	0.04	0.03	+++	0.102	0.08	0.20	++-
rs2033732	8	85079709	C	T	*RALYL*	0.019	0.004	T	C	0.007	0.02	0.72	+-*	0.058	0.11	0.59	+-*	-0.012	0.04	0.77	0-*	-0.010	0.03	0.73	--*	0.009	0.03	0.79	+-*	-0.094	0.08	0.22	--*
rs4740619	9	15634326	T	C	C9orf93	0.018	0.003	T	C	0.010	0.02	0.50	+--	-0.088	0.07	0.19	---	0.016	0.03	0.57	+-+	-0.008	0.02	0.74	++-	0.024	0.02	0.32	+-+	0.006	0.05	0.91	++-
rs1096857(	9	28414339	G	A	*LINGO2*	0.025	0.003	A	G	0.003	0.02	0.86	-++	-0.082	0.07	0.21	+--	0.045	0.03	0.11	+++	0.029	0.02	0.23	+++	-0.027	0.03	0.32	--+	0.025	0.05	0.65	+--
rs6477694	9	111932342	C	T	EPB41L4B	0.017	0.003	T	C	0.004	0.02	0.79	+--	0.053	0.06	0.41	+++	-0.023	0.03	0.39	+--	0.003	0.02	0.88	+--	-0.035	0.03	0.16	---	0.015	0.05	0.78	-++
rs1928295	9	120378483	T	C	*TLR4*	0.019	0.003	T	C	0.015	0.02	0.34	+++	-0.026	0.07	0.71	++-	0.002	0.03	0.95	-++	0.025	0.02	0.28	++-	0.002	0.02	0.92	-+-	0.016	0.05	0.77	-++
rs1073368:	9	129460914	A	G	*LMX1B*	0.017	0.003	A	G	-0.004	0.02	0.80	-+*	-0.070	0.09	0.43	-+*	-0.021	0.03	0.54	--*	-0.039	0.02	0.12	--*	0.029	0.03	0.28	++*	-0.010	0.06	0.87	-+*
rs7899106	10	87410904	G	A	GRID1	0.040	0.007	A	G	0.121	0.04	0.00	++*	0.139	0.26	0.59	++*	0.268	0.09	0.00	++*	0.127	0.05	0.02	++*	0.023	0.06	0.70	+-*	0.080	0.16	0.63	++*
rs1709422:	10	102395440	C	T	*HIF1AN*	0.025	0.004	T	C	-0.014	0.02	0.53	-+*	0.130	0.11	0.25	++*	0.024	0.04	0.59	++*	-0.026	0.03	0.43	-+*	0.003	0.03	0.93	-+*	0.050	0.08	0.52	-+*
rs1119156(	10	104869038	C	T	*NT5C2*	0.031	0.005	T	C	-0.021	0.03	0.43	--+	0.126	0.11	0.26	+-+	0.023	0.05	0.61	+-+	-0.026	0.04	0.49	+--	-0.056	0.04	0.20	--+	-0.034	0.09	0.71	+--
rs7903146	10	114758349	C	T	TCF7L2	0.023	0.003	T	C	-0.025	0.02	0.13	-+-	0.075	0.07	0.28	+-+	-0.042	0.03	0.15	-+-	-0.004	0.02	0.86	-++	-0.015	0.03	0.58	-++	-0.103	0.06	0.08	---
rs4256980	11	8673939	G	C	TRIM66	0.021	0.003	C	G	0.017	0.02	0.27	+++	-0.048	0.06	0.45	-+-	0.048	0.03	0.08	-++	-0.021	0.02	0.35	-++	0.027	0.03	0.29	+-+	0.049	0.05	0.35	+-+
rs1103010	11	27684517	A	G	*BDNF*	0.041	0.004	A	G	0.042	0.02	0.02	+++	0.097	0.08	0.20	-++	0.009	0.03	0.79	-++	-0.006	0.03	0.83	-+-	0.080	0.03	0.00	+++	0.006	0.06	0.92	-++
rs2176598	11	43864278	T	C	*HSD17B12*	0.020	0.004	T	C	-0.005	0.02	0.79	+--	-0.219	0.08	0.00	---	0.007	0.03	0.82	-++	-0.036	0.03	0.17	---	-0.001	0.03	0.97	--+	-0.042	0.06	0.49	-+-
rs3817334	11	47650993	T	C	MTCH2	0.026	0.003	T	C	-0.005	0.02	0.73	+--	-0.154	0.06	0.02	---	-0.005	0.03	0.85	+--	-0.035	0.02	0.12	---	0.025	0.03	0.32	+-+	0.033	0.05	0.53	-++
rs1228692!	11	115022404	G	A	*CADM1*	0.022	0.003	A	G	-0.041	0.01	0.01	--+	0.002	0.06	0.98	+--	-0.035	0.03	0.19	---	-0.039	0.02	0.07	-++	-0.032	0.02	0.18	--+	0.056	0.05	0.28	0++
rs7138803	12	50247468	A	G	BCDIN3D	0.032	0.003	A	G	-0.031	0.02	0.04	---	0.064	0.06	0.31	++-	-0.027	0.03	0.32	---	0.002	0.02	0.94	+--	-0.081	0.02	0.00	---	-0.045	0.05	0.39	--+
rs1105740!	12	122781897	G	A	*CLIP1*	0.031	0.006	A	G	0.007	0.03	0.80	+--	-0.096	0.11	0.38	+--	-0.012	0.04	0.79	--+	-0.032	0.04	0.41	---	0.026	0.04	0.51	++-	-0.196	0.09	0.03	---
rs9581854	13	28017782	T	C	MTIF3	0.030	0.005	T	C	0.005	0.02	0.80	--+	-0.105	0.10	0.29	*+-	0.080	0.05	0.08	*++	-0.028	0.03	0.34	--+	0.005	0.03	0.86	+++	0.167	0.09	0.06	*++
rs1242954!	13	54102206	A	G	*OLFM4*	0.033	0.005	A	G	0.015	0.02	0.52	+--	-0.040	0.09	0.67	+--	0.089	0.04	0.03	+++	-0.019	0.03	0.57	+--	0.065	0.04	0.09	+-+	0.017	0.08	0.83	-+-
rs9540493	13	66205704	A	G	MIR548X2	0.017	0.003	A	G	0.004	0.02	0.81	++*	-0.081	0.09	0.38	-+*	-0.024	0.04	0.50	--*	0.020	0.03	0.47	+-*	-0.012	0.03	0.66	--*	-0.002	0.06	0.98	-+*
rs1441264	13	79580919	A	G	*MIR548A2*	0.018	0.003	A	G	0.011	0.02	0.52	+-*	0.012	0.09	0.90	+-*	-0.011	0.04	0.75	+-*	0.005	0.03	0.84	+-*	-0.003	0.03	0.92	+-*	0.025	0.06	0.70	+-*
rs1013228(	14	25928179	C	A	*STXBP6*	0.023	0.003	A	C	-0.021	0.02	0.25	--*	-0.017	0.11	0.87	+-*	0.010	0.04	0.81	+-*	0.016	0.03	0.58	+-*	0.009	0.03	0.77	+-*	-0.030	0.07	0.68	--*
rs1288545	14	29736838	C	A	*PRKD1*	0.021	0.003	A	C	-0.036	0.02	0.04	-+*	-0.164	0.10	0.10	--*	-0.050	0.04	0.18	-+*	-0.020	0.03	0.45	-+*	-0.023	0.03	0.40	-+*	-0.059	0.07	0.38	--*
rs1184769	14	30515112	T	C	*PRKD1*	0.049	0.008	T	C	0.053	0.04	0.18	+++	-0.074	0.16	0.65	+--	0.095	0.07	0.15	++-	-0.014	0.06	0.81	+--	0.049	0.06	0.43	+++	0.466	0.11	0.00	+++

rs7141420	14	79899454	T	C	NRXN3	0.024	0.003	T	C	-0.006	0.02	0.73	-+*	-0.049	0.09	0.59	+-*	0.027	0.03	0.44	-+*	0.006	0.03	0.81	+-*	-0.017	0.03	0.52	-+*	0.112	0.06	0.08	++*
rs3736485	15	51748610	A	G	DMXL2	0.018	0.003	A	G	0.022	0.02	0.17	+-+	0.083	0.07	0.22	-++	0.034	0.03	0.22	+-+	0.015	0.02	0.54	+++	0.030	0.03	0.25	+--	-0.035	0.05	0.52	+-+
rs1695127	15	68077168	T	C	*MAP2K5*	0.031	0.004	T	C	0.027	0.02	0.13	+0+	-0.058	0.08	0.44	-+-	0.012	0.03	0.71	+-+	0.009	0.03	0.73	++-	0.052	0.03	0.07	+++	0.101	0.06	0.12	+++
rs7164727	15	73093991	T	C	.OC10028755	0.018	0.003	T	C	0.035	0.02	0.03	+++	0.043	0.07	0.53	+-+	0.043	0.03	0.13	+++	0.002	0.02	0.94	++-	0.055	0.03	0.03	+++	0.027	0.06	0.62	++-
rs758747	16	3627358	T	C	NLRC3	0.023	0.004	T	C	0.031	0.02	0.08	+-+	0.182	0.07	0.01	+++	-0.013	0.03	0.66	+--	0.040	0.03	0.13	+++	0.001	0.03	0.96	++-	0.069	0.06	0.25	+-+
rs1244663	16	19935389	G	A	*GPRC5B*	0.040	0.005	A	G	-0.012	0.02	0.57	--+	0.033	0.09	0.72	-++	-0.035	0.04	0.35	+--	-0.020	0.03	0.54	---	-0.091	0.03	0.01	---	0.071	0.07	0.32	+-+
rs2650492	16	28333411	A	G	SBK1	0.021	0.004	A	G	0.021	0.02	0.23	+--	-0.167	0.07	0.02	---	0.018	0.03	0.53	++-	-0.026	0.02	0.30	---	0.046	0.03	0.09	++-	-0.019	0.06	0.73	--+
rs3888190	16	28889486	A	C	ATP2A1	0.031	0.003	A	C	0.035	0.02	0.03	+++	-0.133	0.06	0.04	---	0.046	0.03	0.08	+++	0.004	0.02	0.86	+--	0.069	0.03	0.01	+++	0.038	0.05	0.46	+-+
rs4787491	16	30015337	G	A	INO80E	0.016	0.003	A	G	0.033	0.02	0.03	+++	-0.007	0.06	0.91	-+-	0.065	0.03	0.01	+++	0.016	0.02	0.49	++-	0.040	0.02	0.10	+++	0.047	0.05	0.36	++-
rs9925964	16	31129895	A	G	KAT8	0.019	0.003	A	G	0.027	0.02	0.08	+++	0.015	0.06	0.82	-++	0.018	0.03	0.50	+-+	-0.007	0.02	0.78	-++	0.065	0.02	0.01	+++	0.052	0.05	0.33	+-+
rs2080454	16	49062590	C	A	*CBLN1*	0.017	0.003	A	C	0.023	0.02	0.19	++*	-0.081	0.09	0.38	--*	0.054	0.04	0.14	++*	0.016	0.03	0.55	++*	-0.005	0.03	0.86	--*	0.079	0.06	0.22	++*
rs1558902	16	53803574	A	T	*FTO*	0.082	0.003	A	T	-0.024	0.03	0.41	*+-	-0.058	0.06	0.36	---	-0.025	0.03	0.35	+--	-0.055	0.04	0.17	*--	0.004	0.05	0.93	*-+	-0.004	0.05	0.94	++-
rs9914578	17	2005136	G	C	SMG6	0.020	0.004	C	G	0.019	0.02	0.31	+--	0.072	0.09	0.40	+++	-0.030	0.04	0.39	+--	-0.006	0.03	0.85	+-+	0.035	0.03	0.24	+--	0.060	0.07	0.38	+-+
rs1000940	17	5283252	G	A	*RABEP1*	0.019	0.003	A	G	0.002	0.02	0.91	+--	-0.003	0.07	0.97	-+-	-0.018	0.03	0.54	0--	-0.003	0.02	0.89	++-	-0.002	0.03	0.95	++-	-0.031	0.06	0.58	--+
rs1294062	17	78615571	G	A	*RPTOR*	0.018	0.003	A	G	0.020	0.02	0.24	++*	0.070	0.09	0.44	-+*	-0.071	0.04	0.05	--*	0.029	0.03	0.28	++*	0.021	0.03	0.45	+-*	-0.087	0.06	0.17	-+*
rs1808579	18	21104888	C	T	*C18orf8*	0.017	0.003	T	C	-0.025	0.01	0.09	--+	-0.053	0.06	0.40	---	-0.001	0.03	0.96	+--	-0.007	0.02	0.74	-+-	-0.050	0.02	0.03	-+-	-0.034	0.05	0.51	--+
rs7239883	18	40147671	G	A	LOC284260	0.016	0.003	A	G	-0.002	0.02	0.92	+-*	0.007	0.09	0.94	-+*	-0.053	0.03	0.13	--*	0.002	0.03	0.94	+-*	-0.020	0.03	0.46	--*	-0.051	0.06	0.41	--*
rs7243357	18	56883319	T	G	GRP	0.022	0.004	T	G	0.007	0.02	0.74	+-+	-0.032	0.08	0.70	+-+	-0.014	0.03	0.69	--+	0.015	0.03	0.61	+-+	0.025	0.03	0.42	+-+	-0.072	0.07	0.28	---
rs6567160	18	57829135	C	T	MC4R	0.056	0.004	T	C	-0.027	0.02	0.14	-+-	0.147	0.08	0.06	0++	-0.045	0.03	0.15	-+-	-0.012	0.03	0.65	-+-	-0.034	0.03	0.25	---	-0.134	0.06	0.02	---
rs1772499	19	18454825	A	G	*PGPEP1*	0.019	0.004	A	G	0.039	0.02	0.04	++*	0.190	0.11	0.09	++*	0.028	0.04	0.51	-+*	0.037	0.03	0.20	++*	0.064	0.03	0.02	++*	0.049	0.08	0.52	-+*
rs29941	19	34309532	G	A	KCTD15	0.018	0.003	A	G	-0.003	0.02	0.88	-+-	0.057	0.07	0.45	+++	0.000	0.03	0.99	++-	-0.006	0.02	0.83	++-	-0.027	0.03	0.30	-+-	0.028	0.06	0.63	+-+
rs2075650	19	45395619	A	G	*TOMM40*	0.026	0.005	A	G	0.032	0.02	0.17	+-*	0.019	0.13	0.88	-+*	0.002	0.05	0.97	+-*	0.038	0.04	0.28	+-*	0.011	0.04	0.75	+-*	0.056	0.09	0.53	+-*
rs2287019	19	46202172	C	T	*QPCTL*	0.036	0.004	T	C	-0.002	0.02	0.92	++-	0.092	0.08	0.23	+++	-0.029	0.03	0.37	++-	0.025	0.03	0.39	++-	-0.039	0.03	0.18	-+-	-0.071	0.07	0.28	-+-
rs3810291	19	47569003	A	G	ZC3H4	0.028	0.004	A	G	0.005	0.02	0.78	+--	0.034	0.07	0.62	+++	-0.031	0.03	0.30	-+-	0.010	0.03	0.71	++-	0.004	0.03	0.87	++-	0.009	0.06	0.87	+-+
rs6091540	20	51087862	C	T	ZFP64	0.019	0.004	T	C	0.016	0.02	0.35	+-+	0.032	0.07	0.65	-++	0.001	0.03	0.97	--+	-0.001	0.02	0.97	--+	0.016	0.03	0.56	+-+	0.034	0.06	0.55	-++
rs2836754	21	40291740	C	T	ETS2	0.016	0.003	T	C	0.009	0.02	0.58	+-+	-0.060	0.06	0.35	-+-	-0.003	0.03	0.91	--+	-0.020	0.02	0.41	+--	0.016	0.03	0.53	+--	-0.017	0.05	0.75	+-+

Supplementary Table S8 - BMI causal effects from a likelihood-based MR approach using 96 BMI SNPs.

Sample	Smoking	N Cases	N Controls	OR	LIC	UIC	P	P Het
**Overall**	**All**	16,241	21,164	1.10	0.95	1.27	0.20	8.20E-04
	**Never**	712	3,032	0.47	0.26	0.84	0.01	0.22
	**Ever**	7,049	6,377	1.09	0.86	1.39	0.46	0.01
**AD**	**All**	5,282	21,164	0.82	0.66	1.01	0.06	0.92
**SQ**	**All**	4,224	21,164	1.39	1.10	1.74	0.01	0.01
**SC**	**All**	927	8,833	1.83	1.14	2.92	0.01	0.19

Supplementary Table S9 - Fold risk increase of BMI on lung cancer types provided by weighted median MR approach

Sample	Smoking	N Cases	N Controls	OR	LIC	UIC	P
**Overall**	**All**	16,241	21,164	1.18	0.94	1.48	0.14
	**Never**	712	3,032	0.51	0.17	1.48	0.22
	**Ever**	7,049	6,377	1.09	0.73	1.61	0.69
**AD**	**All**	5,282	21,164	0.93	0.68	1.28	0.67
**SQ**	**All**	4,224	21,164	1.40	0.98	2.02	0.07
**SC**	**All**	927	8,833	1.42	0.66	3.06	0.37

Supplementary Table S10- Overall pleiotropic effect assessment of causal estimates of BMI on lung cancer types provided by MR-Egger test

Sample	Smoking	N Cases	N Controls	Intercept	LIC	UIC	P
**Overall**	**All**	16,241	21,164	0.00	-0.01	0.01	0.56
	**Never**	712	3,032	-0.01	-0.04	0.03	0.81
	**Ever**	7,049	6,377	0.00	-0.02	0.01	0.62
**AD**	**All**	5,282	21,164	0.01	-0.01	0.02	0.41
**SQ**	**All**	4,224	21,164	0.00	-0.02	0.01	0.58
**SC**	**All**	927	8,833	-0.02	-0.05	0.01	0.24

Supplementary Figure S1- Power calculations for MR analysis with an instruments explaining 2.7% of phenotype variance in the sample groups. Overall: Lung cancer overall, AD: Adenocarcinoma, SQ: Squamous cell carcinoma, SC: Small cell carcinoma, Ever: Lung cancer overall ever smokers, Never: Lung cancer overall never smokers.

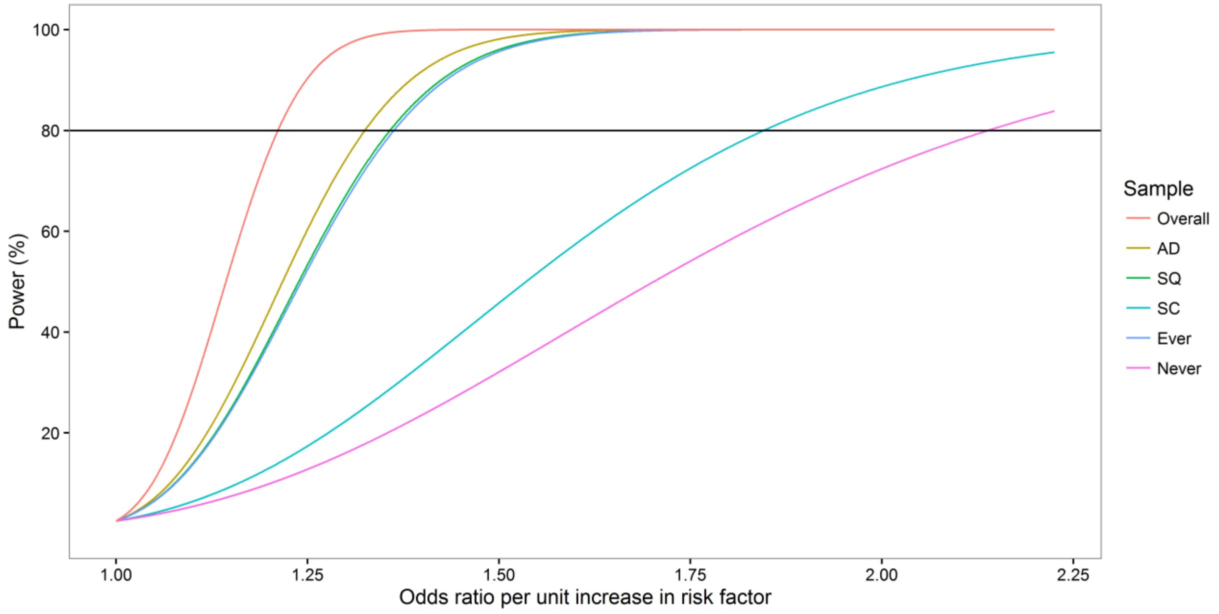

Supplement: Supplementary Information [file srep31121-s1.pdf]
